# Gut microbiota and its metabolism in autism spectrum disorder: from pathogenesis to therapy

**DOI:** 10.3389/fcimb.2025.1687691

**Published:** 2026-01-05

**Authors:** Wanping Bu, Zhichuan Chen, Bo Liu, Xiaokang Jia

**Affiliations:** 1 School of Clinical Medicine, Quanzhou Medical College, Quanzhou, Fujian, China; 2 School of Traditional Chinese Medicine, Hainan Academy of Medical Sciences, Hainan Medical University, Haikou, Hainan, China; 3 Key Lab for Basic Pharmacology and Joint International Laboratory of Ethnomedicine of Ministry of Education, Zunyi Medical University, Zunyi, China

**Keywords:** autism spectrum disorder, FMT, gut microbiota, pathogenesis, therapy

## Abstract

Autism Spectrum Disorder (ASD) is a complex neurodevelopmental disorder characterized by social communication deficits and repetitive behaviors. Studies show that nearly half of ASD patients have gastrointestinal symptoms such as abdominal pain and diarrhea, indicating the important role of gut microbiota in its pathogenesis. This review finds that ASD patients exhibit reduced gut microbiota diversity and imbalanced Bacteroidetes/Firmicutes ratio, with abnormal microbial structure affecting neurobehavior through the gut-brain axis. Abnormalities in gut microbiota metabolites (short-chain fatty acids, phenolic compounds, bile acids, amino acids, etc.) are key mediators, which can exacerbate symptoms by affecting BBB permeability, neuroinflammation, and neurotransmitter balance. The gut-brain axis regulates ASD through mechanisms including the HPA axis, vagus nerve, immune pathways, and barrier functions. Gut microbiota-targeted interventions (exercise, dietary intervention, fecal microbiota transplantation, prebiotics/probiotics, etc.) can alleviate gastrointestinal and behavioral symptoms of ASD by regulating microbiota balance and improving metabolic environment. However, there are still issues such as unclear metabolite regulation mechanisms and significant individual differences in interventions. Future studies should combine multi-omics and artificial intelligence to identify core targets, develop personalized plans, and promote clinical translation.

## 1 Introduction

Autism Spectrum Disorder (ASD) is a complex neurodevelopmental disorder primarily characterized by deficits in social interaction and communication, delayed language development, restricted interests, and repetitive behaviors. According to recent epidemiological data from the United States, the prevalence of ASD is approximately 2.3% (Hirota and King, 2023). In China, the estimated prevalence of ASD among children aged 6 to 12 years is 0.70% (Zhou et al., 2020), and this prevalence is expected to continue rising over time (Elsabbagh et al., 2012; Zeidan et al., 2022). Notably, up to 48.67% of patients exhibit gastrointestinal complications such as abdominal pain, diarrhea, constipation, and gastroesophageal reflux, suggesting the significant role of the gastrointestinal system and its microbiota in the onset and progression of ASD (Wang et al., 2022).

Furthermore, gastrointestinal physiology in ASD patients is often abnormal, including increased intestinal permeability and alterations in the overall microbiome (Mandy and Lai, 2016; Chatterjee et al., 2023; Mathew et al., 2024). The pathogenesis of ASD is also associated with changes in the gut microbiota of both the mother and the individual. Studies have indicated that alterations in maternal gut microbiota may increase the risk of ASD in offspring. Compared to healthy children, children with autism exhibit reduced gut microbiota diversity, and their microbiota developmental trajectory deviates from that of neurotypical (NT) populations (Li et al., 2019). These findings further support the direct relationship between gut microbiota and ASD.

With the advancement of sequencing technologies, the role of gut microbiota and its metabolites in modulating ASD has been partially elucidated. Increasing numbers of observational studies have explored the potential of modulating gut microbiota to alleviate ASD-related symptoms. The aim of these studies is to explore gut microbiota as a breakthrough point to expand the intervention strategies for ASD.

This review will focus on the relationship between gut microbiota dysbiosis and ASD, the impact of the gut-brain axis on the pathogenesis of ASD, and potential therapeutic approaches targeting the gut microbiota, with the hope of uncovering the underlying mechanisms of ASD and providing additional intervention strategies for its treatment.

## 2 Gut microbiota and ASD

### 2.1 Dysregulated gut microbiota in patients with ASD disorder

Studies have shown that 48.67% of individuals with ASD have gastrointestinal symptoms (Wang et al., 2022), and the incidence of gastrointestinal symptoms in children with ASD is four times higher than that in the general population. Common symptoms include constipation, diarrhea, bloating, abdominal pain, reflux, vomiting, flatulence, foul-smelling stools, and food allergies (Fulceri et al., 2016; Santocchi et al., 2016; Iovene et al., 2017; Marler et al., 2017). An increasing body of evidence suggests that gut microbiota dysbiosis in ASD patients is closely associated with gastrointestinal symptoms, and the severity of these symptoms is strongly correlated with the clinical manifestations of ASD (Kreider et al., 2021). Specifically, there is a clear connection between the gut microbiota of ASD patients and their neurobehavioral symptoms. Studies by Deng, W (Deng et al., 2022)and Liu, J (Liu et al., 2022)have found that gastrointestinal symptoms, such as diarrhea and constipation, may exacerbate behavioral issues in children with ASD, such as rigidity, hyperactivity, and social withdrawal. Animal studies by Sharon G (Sharon et al., 2019)and others have shown that when the gut microbiota from ASD patients is transplanted into GF mice, the mice exhibit symptoms similar to those of ASD, further supporting the link between gut microbiota and the pathogenesis of ASD. Research also indicates that ASD patients exhibit abnormalities in gut microbiota in terms of biomass, biodiversity, richness (Ma et al., 2019; Chang et al., 2024), and structure (Chang et al., 2024). To better understand this phenomenon, we have summarized six relevant studies from five countries over the past three years (see **Table 1**). As shown in **Table 1**, the gut microbiota of ASD patients differs significantly from that of the general population, and these patients’ gut microbiota undergoes noticeable changes. Although these studies have not consistently identified specific microbial species that change, this may be due to differences in factors such as diet, age, sex, population, and the severity of autism (Pulikkan et al., 2018). Nonetheless, the studies consistently suggest that ASD patients often exhibit microbial imbalances of various types. The most evident change is the decreased ratio of Bacteroidetes/Firmicutes, which may reflect the reduced relative abundance of Bacteroidetes (Alharthi et al., 2022), though this shift could also be influenced by confounding factors such as medication use.

**Table 1:** Summary of studies on gut microbiota alterations in ASD patients.

Authors	Year	Country	Research method	Sample type	Reduced microbial abundance	Increased microbial abundance	Key findings	Reference type	Research design and methodology	Exclusion criteria	Inclusion criteria
(Chang et al., 2024)	2024	China	Metagenomic sequencing	Fecal samples from ASD subjects and matched normal children	Class-level *Deinococci* and *Holophagae* significantly reduced (first reported), the levels of Prostrata, invisible Dialister and Bacteroides decreased	No significantly increased microbiota mentioned; *Thermococci* abundance significantly decreased; the abundance of inosine, glutamic acid, xanthine and methylxanthine increased.	Children with ASD had lower richness and higher evenness of microbiota, and the structure of microbiota was changed.	Clinical study	Non-randomized controlled studies	1. A history of congenital diseases. 2. Presence of acute or chronic affective disorders within the past three months. 3. No use of antibiotics, probiotics, prebiotics, or other agents that may influence intestinal microbiota during the three-month period preceding fecal sample collection.	Thirty Chinese children (20 males and 10 females) aged 2-4 years diagnosed with ASD and 30 age-and gender-matched normal children were enrolled
(Li et al., 2024)	2024	China	Metagenomic sequencing	Fecal samples from Chinese children with ASD, and normal children.	Levels of Prevotella, Dialister invisus, and Bacteroides were decreased.	The abundance of inosine, glutamate, xanthine, and methylxanthine was increased.	The gut bacterial community and bacteriophage population exhibit a high degree of symbiotic interaction. The combination of Probio-M8 supplementation and a moderate-carbohydrate diet may effectively alleviate symptoms associated with ASD.	Clinical study	Prospective study (single-arm and open-label design)	1) Diagnosis of malnutrition; 2) Presence of severe gastrointestinal disorders necessitating immediate treatment; 3) Use of immunosuppressive agents, antibiotics, probiotics, prebiotics, or synbiotics within one month prior to the intervention; 4) Occurrence of severe fever or active infection within seven days before enrollment; 5) Renal insufficiency or impaired liver function; 6) Known hypersensitivity or allergy to probiotics or any components included in the study intervention.	A total of 72 Chinese children aged 3 to 12 years who were diagnosed with ASD, irrespective of gender, were included in the study. Additionally, data from 29 TD Chinese children sourced from the National Center for Biotechnology(NCBI) database and 16 locally recruited TD Chinese children were also analyzed.
(Chamtouri et al., 2023a)	2023	The Republic of Tunisia	16S rRNA sequencing	Fecal samples from Tunisian children with ASD, age-matched ASD children, siblings and normal children	Key features of the microbiota composition associated with Bifidobacterium abundance	ASD patients have significantly higher levels of PPA and valeric acid than General Practitioner (GP) patients at the age of 4 to 7, but these differences disappear at the age of 8 to 10	The changes of gut microbiota in the early stage of ASD are more obvious than in the late stage, and the effect of early intervention on neurological symptoms and gastrointestinal symptoms is better than that in the late stage.	Clinical study	A meta-taxonomic approach	Infections, other neurological disorders not strictly associated with autism, type 1 diabetes, genetic syndromes, unbalanced or special diets, celiac disease, food intolerances, and inflammatory bowel disease were excluded. Additionally, the subjects in this study had not received antibiotic or antifungal treatment, nor had they taken probiotics and/or prebiotics for at least one month prior to sampling.	The study included 74 Tunisian children aged 4 to 10 years with a clinical diagnosis of ASD, along with 18 age-matched siblings of ASD children and 28 age-matched TD children.
(Novau-Ferré et al., 2025)	2025	Spain	16S rRNA sequencing	Fecal samples from Spanish children aged 5-14 years and clinically diagnosed with ASD	The abundance of Eggertella, Ruminococcus and Clostridium decreased.	Bacteroides, Bacillus and actinomycetes	Unique characteristics of the gut microbiota in children with ASD were identified, specifically the increased abundances of Bacteroidetes, Bacilli, and Actinobacteria.	Clinical study	A 12-week randomized, double-blind, placebo-controlled trial.	Having taken probiotics in the 3 months prior to the study, being under antibiotic treatment, having intolerance or allergic reactions to the excipients of the treatment, and any medical conditions incompatible with the intervention.	Forty-one Spanish children aged between 5 and 14 years, with a clinical diagnosis of ASD.
(Hrnciarova et al., 2024)	2024	Czech Republic	16S rRNA sequencing	Czech children with Fecal samples from ASD and NT children.	Firmicutes	Actinobacteria and Proteobacteria	The intestinal flora characteristics of patients with mild ASD symptoms and those with severe ASD symptoms are different. The abundance of Bacteroides is higher in cases with milder ASD symptoms. The most severe symptoms in children with ASD include Prevotella, Escherichia/Shigella, Veillonella, Streptococcus, Alistipes and Bifidobacterium is relatively high.	Clinical study	A three-month prospective, double-blind, randomized study	Not mentioned	Sixteen Czech children aged 3 to 7 who were clinically diagnosed with ASD and 12 children with NT conditions.
(Kadiyska et al., 2025)	2025	The Republic of Bulgaria	16S rRNA sequencing	Fecal samples from Bulgarian children and adolescents diagnosed with ASD, a previously reported meta-analysis of European NT populations of the same age group.	Actinobacteria decreased	Proteobacteria increased and endotoxin-producing bacteria (Proteobacteria) were relatively enriched	The dysbiotics of gut microbiota can occur in all age stages of ASD, which is dynamic, but the most significant is 0-4 years old.	Clinical study	Fecal DNA was extracted from 302 Bulgarian children and young adults aged 2-19 years and the gut microbiota data were analyzed and compared with the mean of a meta-analysis of European NT populations of the same age group reported previously.	Antibiotics, probiotics, or prebiotics were administered within 4 weeks prior to the study	302 Bulgarian children and adolescents aged 2-19 years diagnosed with ASD.

### 2.2 How gut microbiota dysbiosis contributes to autism spectrum disorder

**Figure 1:**
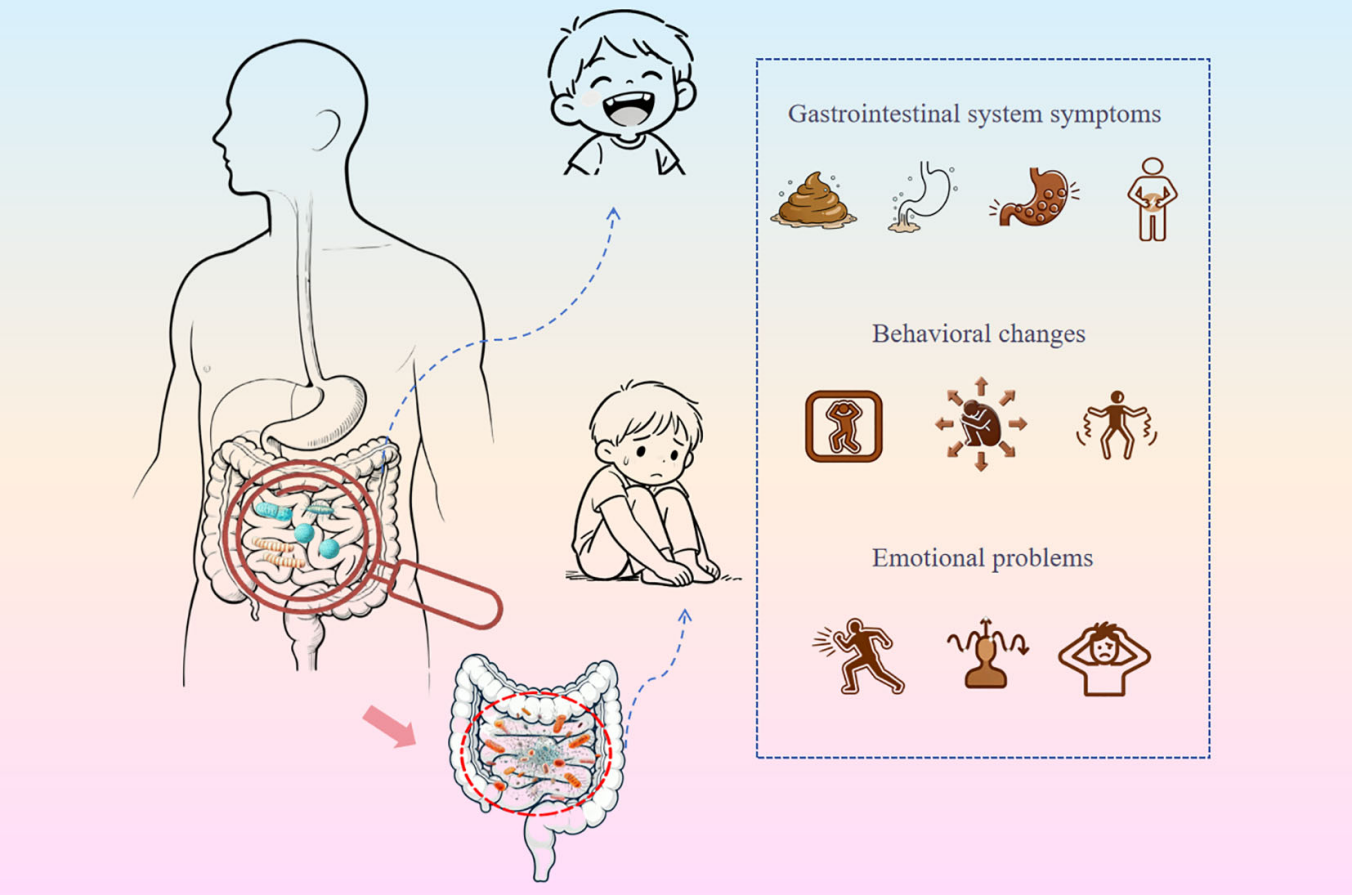
Dysregulated gut microbiota in patients with ASD. This diagram visually illustrates the core concept of the gut-brain axis: gastrointestinal symptoms (e.g., digestive issues caused by gut microbiota dysbiosis) directly influence brain function through bidirectional communication along this axis, triggering behavioral changes (e.g., exacerbated stereotyped behaviors in ASD patients) and emotional problems (e.g., anxiety and depression), while these neurobehavioral manifestations in turn exacerbate gastrointestinal symptoms, forming a vicious cycle.

Human gut microbiota colonization begins during the fetal period and matures to a composition close to that of adults by around 3 years of age—a critical window that overlaps with the golden period of childhood brain development (Collado et al., 2016; Margolis et al., 2021). Emerging evidence indicates that gut microbiota can exert significant influences on brain function (Damiani et al., 2023). Dysbiosis of gut microbiota is closely linked to the onset and progression of ASD, exerting impacts on both gastrointestinal symptoms and neurobehavioral manifestations through microbiota-gut-brain axis-related mechanisms. Specifically, gut microbiota dysbiosis alters microbial metabolites and disrupts the intestinal microenvironment, establishing bidirectional communication between the gut and the brain through integrated effects on the nervous, immune, and endocrine systems, ultimately modulating brain function (Chernikova et al., 2021; Socala et al., 2021) (**Figure 1**).

In animal experiments, Sharon G et al. transplanted gut microbiota from ASD patients or typically developing (TD) controls into GF mice, demonstrating that colonization with ASD-derived microbiota was sufficient to induce hallmark autistic-like behaviors (Sharon et al., 2019). They detected reduced levels of specific metabolite profiles—particularly 5-aminovaleric acid (5AV) and taurine—in the ASD group, proposing that gut microbiota regulate murine behavior by producing neuroactive metabolites (Sharon et al., 2019). Moreover, ASD-associated microbiota promote widespread alternative splicing of ASD-related genes in the brain (Stilling et al., 2018), and the gut microbiome can alter splicing programs at the level of ribosomal binding proteins (RBPs) (Sharon et al., 2019).

De Angelis and colleagues noted that gut microbiota dysbiosis is commonly associated with impairment of the intestinal mucosal barrier, which increases intestinal permeability to neurotoxic compounds derived from diet or gut bacterial metabolism. This disruption leads to dysregulation of neuromodulatory mechanisms and impairment of normal brain development (De Angelis et al., 2015). Additionally, accumulating research confirms that alterations in gut microbial composition are linked to immune dysfunction in ASD patients. Gut microbiota can indirectly influence the innate immune system, thereby altering circulating levels of pro-inflammatory and anti-inflammatory cytokines and impacting microglial homeostasis (Erny et al., 2015; Alharthi et al., 2022).

In summary, a balanced gut microbial composition is essential for maintaining microbial homeostasis, whereas perturbations in microbial composition can exert adverse effects on human health, contributing to the pathogenesis and progression of ASD.

## 3 Gut microbiota-derived metabolites and autism spectrum disorder

Gut microbiota-derived metabolites may play a pivotal role in regulating the pathogenesis of ASD. Studies by Sharon, G et al. have confirmed that the production of microbial metabolites in the gut can affect brain function and modulate behavioral phenotypes in ASD patients (Sharon et al., 2019), while small-molecule metabolites are capable of precisely regulating gene expression, Ribonucleic Acid(RNA) splicing, and neuronal function in the brain (Nankova et al., 2014). Scholars have proposed that gut microbiota-derived metabolites may even act as core drivers of systemic inflammation and subsequent neuroinflammation (Panelli et al., 2020). Alterations in gut microbiome structure typically trigger metabolic profile disorders, thereby affecting the availability and diversity of nutrients and microbial metabolites (Dodd et al., 2017; Sharon et al., 2019). As observed in metabolomic analyses of serum, feces, and urine from ASD subjects by De Angelis (De Angelis et al., 2013)’s team and Wang M (Wang et al., 2019) et al., numerous molecules in ASD patients show significant differences compared to TD individuals, with a large number of these dysregulated compounds originating from metabolic processes of gut microorganisms. Animal experiments by Lu Xiao et al (Xiao et al., 2021). further validated this association, revealing that the gut microbiome of ASD children can induce ASD-like behavioral characteristics in germ-free (GF) mice through metabolites.

Notable abnormalities in gut microbiota-derived metabolites include: functional disorders in amino acid transport and degradation pathways; significantly elevated levels of p-cresol and 4-ethylphenol (4EP), which correlate with symptom severity in ASD patients; imbalanced bile acid metabolism, characterized by reduced levels of secondary bile acids and accumulation of conjugated bile acids; metabolic abnormalities in SCFAs (such as excessive propionate accumulation and relative butyrate deficiency); and dysregulation of tryptophan metabolism pathways, leading to increased kynurenine/tryptophan ratios and insufficient serotonin production (Macfabe, 2012; Golubeva et al., 2017; Settanni et al., 2021; Xiao et al., 2021).

### 3.1 Amino acids

Amino acids serve as precursors for various potent neuroactive molecules (such as classical neurotransmitters), with their metabolites including tryptophan, 5-hydroxytryptamine (5-HT), Gamma-Aminobutyric Acid (GABA), glutamate, and dopamine (DA). 5-HT supports the development of the enteric nervous system (ENS) by activating intestinal peristalsis and electrolyte secretion (Heredia et al., 2013; Mawe and Hoffman, 2013). Studies have revealed insufficient intestinal 5-HT production and reduced bioavailability in ASD patients (Golubeva et al., 2017), accompanied by evidence of abnormal 5-HT metabolism (Lim et al., 2016; Muller et al., 2016). Furthermore, 5-HT metabolites can alter intestinal vagal afferent activity, potentially directly impacting vagal nerve signaling to the brain (Slattery et al., 2006).

Alterations in the levels of several amino acids and their derived metabolites in the feces and/or plasma of ASD patients are closely associated with ASD severity (Al-Otaish et al., 2018; Needham et al., 2021). In their research, Sharon, G et al. compared colonic contents and serum samples between ASD mice and TD mice, identifying 27 significantly differential metabolites among 313 detected compounds in ASD mice’s colonic contents, with generally elevated amino acid concentrations (Sharon et al., 2019). A study by Chang, X et al. further revealed associations between amino acid biosynthesis and neurodegeneration (Chang et al., 2024). Research by Zheng, Y, Kang, D.W, and Chamtouri, M et al. has indicated that altered levels of fecal ammonium salts and various amino acid metabolites (such as p-cresol and 4EP)) are associated with ASD (Kang et al., 2018; Zheng et al., 2021; Chamtouri et al., 2023b).

Observational studies by Mariem Chamtouri et al. found significantly increased total amino acid content and levels of 14 specific amino acids in the feces of ASD children, with a higher glutamate ratio compared to healthy children, which may be related to imbalanced intestinal transamination reactions associated with ASD (Chamtouri et al., 2023b). Studies by Lu Xiao et al (Xiao et al., 2021). confirmed the presence of tryptophan metabolism disorders in ASD patients, characterized by decreased plasma tryptophan levels and an elevated kynurenine/tryptophan ratio. Kynurenine and kynurenic acid can induce increased interleukin-6 (IL-6) levels (Savitz et al., 2015), and related studies have confirmed that IL-6 is one of the most significantly elevated cytokines in ASD patients (Saghazadeh et al., 2019; Filippova et al., 2022; Williams et al., 2022). Under physiological conditions, kynurenic acid exhibits neuroprotective effects, but abnormal elevation of this substance may produce neurotoxicity (Erhardt et al., 2017). Therefore, kynurenic acid, kynurenine, and IL-6 hold promise as potential biomarkers for ASD diagnosis.

### 3.2 Phenolic compounds

P-cresol and 4EP, primarily derived from the metabolism of dietary tyrosine by gut microbiota (Persico and Napolioni, 2013), exhibit abnormal levels in ASD patients that are closely associated with clinical symptoms. P-cresol and its metabolite p-cresyl sulfate are known uremic toxins that can exert negative effects on multiple brain functions (Pascucci et al., 2020). Gevi, F et al. detected significantly elevated levels of p-cresol and p-cresyl sulfate in blood, urine, and fecal samples from ASD patients (Gevi et al., 2020), and proposed that increased levels of these substances are associated with exacerbation of repetitive stereotyped behaviors, communication impairments, and cognitive deficits. Animal experiments by Tiziana Pascucci et al. further confirmed that p-cresol can acutely induce autistic-like behaviors and cause abnormal DA metabolism in reward circuits (Pascucci et al., 2020). Notably, elevated urinary p-cresol levels in autistic children are associated with chronic constipation, suggesting that prolonged intestinal transit time may be one of the main factors contributing to increased intestinal absorption of potential neuroactive compounds such as p-cresol (Gabriele et al., 2016).

High concentrations of 4EP and its metabolite 4-ethylphenyl sulfate (4EPS) in human urine, plasma, or feces are closely linked to autism-related neural and behavioral changes (Stewart et al., 2022; Day et al., 2023). Needham, B.D et al. found through experiments with bioengineered gut bacteria that mice exposed to 4EPS exhibit anxiety-like behaviors, potentially through effects on oligodendrocyte function and myelin patterns in the brain (Needham et al., 2022). Studies by Hsiao, E.Y et al. also confirmed a potential association between elevated levels of 4EPS in plasma and urine of ASD patients and anxious and irritable behaviors (Hsiao et al., 2013). Accumulation of 4EPS in the brain can disrupt functional connectivity between regions such as the hippocampus, thalamus, amygdala, hypothalamus, and cortex (Needham et al., 2022), which are all involved in regulating anxiety behaviors and responses to stress stimuli (Goode et al., 2019; Fischer, 2021).

### 3.3 Bile acids

Bile acids are a group of hydroxylated steroid acids that serve as the main component of bile. They not only facilitate the digestion of dietary lipids and regulate lipid metabolism but also play a crucial role in maintaining epithelial barrier function in the small intestine (Inagaki et al., 2006; Gadaleta et al., 2011). A dynamic bidirectional regulatory relationship exists between the intestinal microbial community and bile acids: the microbiota can precisely regulate the metabolism and synthesis of bile acids, while the composition and size of the bile acid pool in turn influence the diversity and homeostasis of the intestinal microbiota (Guo X. et al., 2022).

Studies have shown that there are defects in the bacterial transformation of bile acids in the intestines of ASD patients. Animal experiments by Golubeva, A V et al. revealed that ASD model mice exhibit significantly reduced plasma bile acid levels and deficient ileal bile acid signaling; simultaneously, the content of all conjugated bile acids in the feces of model mice was significantly increased, while the secondary bile acid pool was markedly reduced, indicating severe bile acid loss (Golubeva et al., 2017). It is known that bile acids can activate colonic peristalsis by stimulating the release of intestinal serotonin and calcitonin gene-related peptide calcitoningene-related peptide(CGRP) (Alemi et al., 2013). Therefore, reduced levels of secondary bile acids lead to delayed colonic transit, which further exacerbates gastrointestinal symptoms such as constipation in ASD patients. In addition, slowed intestinal transit time is one of the important reasons for increased intestinal absorption of more potential neuroactive compounds in ASD patients (Gabriele et al., 2016), a process that may be closely related to impaired intestinal barrier function.

### 3.4 Short-chain fatty acids

Short-chain fatty acids (SCFAs) are metabolites produced by gut bacteria through the fermentation of dietary fiber. As the most abundant bacterial metabolites in the human colon, approximately 95% of SCFAs can be absorbed by the intestine and utilized by the human body, mainly including acetate, propionate, and butyrate (Chen et al., 2017). Evidences have shown that SCFAs exert effects on the brain through two pathways: activating G Protein-Coupled Receptor 41(GPR41) (i.e., free fatty acid receptor 3, which is highly expressed in the brain and blood-brain barrier), and inhibiting histone deacetylase (HDAC) in a dose-dependent manner (Falomir-Lockhart et al., 2019; Guo C. et al., 2022).

SCFAs play multiple regulatory roles in the gut microbiota-gut-brain axis. They can influence the function of the intestinal immune system by regulating gene expression (Iglesias-Vazquez et al., 2020), and can also be absorbed by the central nervous system (CNS) to participate in maintaining the integrity of the blood-brain barrier (BBB), supporting brain development, regulating homeostasis, and modulating neuroinflammation (Silva et al., 2020; Wenzel et al., 2020; Settanni et al., 2021). Clinical studies have found that the content of certain SCFAs in the feces of children with ASD is higher than that in healthy children (Wang et al., 2012; Lagod and Naser, 2023). Research by Xiao, L et al. confirmed that abnormal metabolism of specific SCFAs affects the permeability of the BBB in ASD patients, thereby exerting adverse effects on the neurodevelopment and/or function of their central nervous system (Xiao et al., 2021); in addition, such metabolic abnormalities may lead to changes in mitochondrial function and interfere with the epigenetic regulation of ASD-related genes (Settanni et al., 2021).

Propionic acid (PPA), a short-chain fatty acid produced by anaerobic gut bacteria such as Clostridium and Propionibacterium, has been shown to induce various behavioral, immune, and mitochondrial effects in rodent models highly similar to human autism spectrum disorders (Macfabe, 2012). The concentration of PPA in the feces of ASD children is significantly increased (Coretti et al., 2018), and excessive PPA may have negative impacts on health and behavior through multiple mechanisms, becoming a potential inducer of ASD pathogenesis (Al-Owain et al., 2013). Studies have further confirmed that high concentrations of PPA can cross the BBB and directly damage neurons (Choi et al., 2018), and are closely associated with neurological diseases such as autism (Al-Lahham et al., 2010). Animal experiments have also shown that intracerebroventricular injection of PPA can induce ASD-like behaviors, and its underlying mechanism may be related to abnormal neurotransmitter regulation (Mitsui et al., 2005; MacFabe et al., 2007; Macfabe, 2012). Therefore, reducing PPA exposure and regulating the balance of intestinal microbiota may help reduce the risk of ASD or improve related symptoms.

Butyrate can promote neuroplasticity and memory formation (Stilling et al., 2016), influence social behavior by regulating GABA signaling, and enhance social ability in autistic mouse models. Studies have shown that long-term butyrate treatment can improve ASD-related symptoms (Kratsman et al., 2016). However, the content of butyrate in the feces of ASD children is generally lower than that in healthy children (Zhang et al., 2018; Liu et al., 2019), which may exacerbate social behavior deficits in ASD children by affecting the balance of excitatory/inhibitory systems (Kratsman et al., 2016). Nevertheless, increased butyrate concentrations in fecal samples of ASD patients have also been observed in different studies (Wang et al., 2012; Coretti et al., 2018). Animal experiments indicate that the effects of butyrate on brain development and function are dose-dependent, and high-dose butyrate may induce stress-like responses (Gagliano et al., 2014). In addition, as an anti-inflammatory short-chain fatty acid, butyrate protects colonic health and has been proven to protect ASD cells under oxidative stress and enhance mitochondrial function under physiological stress; meanwhile, butyrate can regulate the synthesis of neurotransmitters such as DA, norepinephrine, and epinephrine, as well as the expression of neurotransmitter genes and ASD-related genes in cell line models (Nankova et al., 2014).The heterogeneity observed in these findings may stem from methodological variations (e.g., fecal vs. plasma detection), cohort characteristics (e.g., age/dietary habits/clinical phenotypes), biological context dependencies (e.g., dose-effect relationships), and sample collection and processing protocols. For instance, discrepancies in the timing, site, and handling of samples across studies can significantly influence results, as gut microbial metabolic activities may fluctuate throughout the day. Additionally, factors such as storage temperature and duration during sample processing can affect the stability of gut microbiota metabolites, thereby introducing deviations in detection outcomes. Notably, in ASD subgroups with constipation, prolonged colonic transit time may lead to localized elevation of butyrate concentrations while reducing systemic absorption. When constipation coexists with other symptoms such as food allergies, further disruption to microbial metabolism may occur. This compartmentalized distribution of metabolites could partially explain contradictory results across different studies (**Table 2**).

## 4 The relationship between the microbiota-gut-brain axis and autism spectrum disorder

Accumulating evidence indicates that gut microbiota, as an environmental factor, can extensively participate in regulating brain functions related to behavior, emotion, and cognition through the microbiota-gut-brain axis. Gut microorganisms can influence central nervous system function via the “gut-brain axis” through immune, neuroendocrine, neurotransmitter, and metabolic pathways (Fairbrass et al., 2022). In this article, we summarize several potential pathways through which the microbiota-gut-brain axis regulates the development of ASD (**Table 2**).

**Table 2:** Relationship between abnormal gut microbiota metabolites and ASD.

Metabolite category	Specific metabolite	Direction of abnormality	Core regulatory pathway/mechanism	Clinical evidence (human studies)	Experimental evidence (animal/cell studies)	Association with ASD symptoms
Amino Acid Metabolism	Tryptophan	Decreased	Abnormal tryptophan metabolism → Elevated kynurenine/tryptophan ratio → Induced release of pro-inflammatory factors (e.g., IL-6) → Neuroinflammation	(Xiao et al., 2021): Reduced plasma tryptophan and elevated kynurenine/tryptophan ratio in ASD patients	(Sharon et al., 2019): Generally elevated amino acid concentrations in colonic contents of ASD model mice	Positively correlated with social withdrawal, repetitive behavior severity
	Glutamate	Increased	Imbalanced intestinal transamination → Glutamate accumulation → Impaired excitatory neurotransmission in gut-brain axis	(Chamtouri et al., 2023b): Higher glutamate ratio in feces of ASD children, associated with intestinal metabolic abnormalities	No direct animal experiments, but *in vitro* studies show glutamate imbalance affects neuronal activity	May exacerbate stereotyped behaviors and irritability
	5-HT	Decreased	Insufficient enteric 5-HT → Impaired ENS development → Disrupted gut-brain axis signaling	Studies found insufficient intestinal 5-HT production and reduced bioavailability in ASD patients	No direct animal experiments, but 5-HT-deficient mouse models show ASD-like intestinal motility abnormalities	Associated with gastrointestinal symptoms (e.g., constipation) and social deficits
Phenolic Compounds	p-Cresol	Increased	Acts as uremic toxin → Impairs BBB integrity → Disturbs central nervous system function	(Gevi et al., 2020): Significantly elevated levels of p-cresol and p-cresyl sulfate in blood, urine, and feces of ASD patients	(Pascucci et al., 2020): p-Cresol induces autistic-like behaviors in mice and causes abnormal DA metabolism in reward circuits	Positively correlated with repetitive stereotyped behaviors, communication impairments, and cognitive deficits
	4EP	Increased	Metabolite 4EPS → Affects oligodendrocyte function → Abnormal myelination → Impaired brain region connectivity	(Needham et al., 2022): Elevated 4EPS levels in plasma and urine of ASD patients, associated with anxiety-like behaviors	(Needham et al., 2022): Mice exposed to 4EPS exhibit anxiety-like behaviors and abnormal functional connectivity in hippocampus, thalamus, etc.	Positively correlated with anxiety and irritable behaviors
Bile Acid Metabolism	Secondary Bile Acids	Decreased	Insufficient secondary bile acids → Delayed colonic transit → Prolonged intestinal transit time → Increased absorption of neurotoxic compounds	(Golubeva et al., 2017): Markedly reduced secondary bile acid pool in feces of ASD model mice, accompanied by abnormal intestinal motility	(Golubeva et al., 2017): Ileal bile acid signaling deficiency in ASD model mice leads to delayed colonic transit and reduced fecal water content	Positively correlated with severity of gastrointestinal symptoms (e.g., constipation)
	Conjugated Bile Acids	Increased	Accumulation of conjugated bile acids → Impaired intestinal barrier function → Release of pro-inflammatory factors → Activation of gut-brain axis inflammation	Ibid., significantly increased conjugated bile acids in feces of model mice	Ibid., bile acid metabolic defects associated with increased intestinal barrier permeability	Indirectly exacerbates neuroinflammation and behavioral abnormalities
SCFAs	PPA	Increased	Excessive PPA → Crosses BBB → Directly damages neurons → Mitochondrial dysfunction and epigenetic regulation disorders	(Wang et al., 2012): Significantly elevated fecal PPA concentrations in ASD children	(MacFabe et al., 2007): Intracerebroventricular injection of PPA induces ASD-like behaviors in rats, related to abnormal neurotransmitter regulation	Positively correlated with hyperactivity, stereotyped behaviors, and cognitive impairment
	Butyrate	Decreased (in most studies)	Butyrate deficiency → Dysregulated GABA signaling → Imbalanced excitatory/inhibitory system → Social behavior deficits	Most studies indicate that children with ASD have lower fecal butyrate levels compared to healthy children; however, some research findings are contradictory.	Buffington et al.: Long-term butyrate treatment improves social ability in autistic mouse models and regulates neuroplasticity	Positively correlated with social withdrawal and emotional regulation deficits

### 4.1 Hypothalamic-pituitary-adrenal axis

The hypothalamic-pituitary-adrenal (HPA) axis is a stress-responsive system in the human body (**Table 3**). When activated, neurotransmitters such as corticotropin-releasing hormone are released in the brain, exerting effects on brain function (Borodovitsyna et al., 2018). Chronic stress can lead to abnormalities in brain structure and functional connectivity, impairing language cognition, sensory perception, and other brain functions, thereby contributing to mental disorders (Dinan and Cryan, 2017). Sudo and colleagues found that the absence of gut microbiota affects the HPA axis, altering endocrine responses to stress (Sudo et al., 2004). Further studies have confirmed that gut microbiota may influence social behaviors in ASD children by participating in discrete neuronal circuits involved in brain stress responses (Wu et al., 2021). Cortisol, the primary HPA axis hormone secreted by the adrenal cortex, affects metabolism, cognition, and behavior (Gao et al., 2022); it also mediates synaptic pruning by activating microglia, thereby influencing the excitatory/inhibitory imbalance in the central nervous system (Gao et al., 2022). Clinical evidence shows significant abnormalities in HPA axis-related hormones in ASD (Singh et al., 2017; Ames et al., 2020; Worsham et al., 2021). Alterations and imbalances in the HPA axis of ASD patients result in significantly elevated cortisol levels in peripheral blood (Spratt et al., 2012). Thus, peripheral cortisol may be partially responsible for the development of ASD (Cornell et al., 2022). Additionally, scholars have proposed an association between maternal premenstrual syndrome and/or postnatal environmental stress factors and the risk of ASD in offspring (Manzari et al., 2019), and maternal cortisol levels are correlated with the developmental trajectory of cognitive function in full-term infants at one year of age (Davis and Sandman, 2010).

**Table 3:** Mechanisms linking the microbiota-gut-brain axis to ASD.

Regulatory pathway	Core abnormal changes	Molecular mechanism details	Experimental evidence (animal/cell studies)	Specific impacts on ASD	Potential intervention targets
HPA Axis	Elevated cortisol levels; Abnormal chronic stress responses	Elevated cortisol → Activates microglia → Abnormal synaptic pruning; HPA axis imbalance → Excitatory/inhibitory imbalance in CNS	(Sudo et al., 2004): Abnormal HPA axis function and enhanced stress response in GF mice; (Wu et al., 2021): Microbiota regulates stress-related neurons affecting social behavior	Impaired social interaction; Cognitive deficits; Emotional regulation disorders	Cortisol receptor antagonists; Probiotics regulating HPA axis activity
Neuroanatomical Pathway (Vagus Nerve)	Impaired vagal signaling; Abnormal development of ENS	Vagal dysfunction → Disrupted gut-brain bidirectional communication; Abnormal ENS motor circuit development → Delayed intestinal transit	(Golubeva et al., 2017): Delayed intestinal transit and colonic elongation in ASD model mice (BTBR strain); (Kim et al., 2013): Impaired small intestinal peristalsis in ASD rat model	Gastrointestinal symptoms (constipation, bloating); Emotional and behavioral abnormalities (anxiety, irritability)	Vagus nerve stimulation; Dietary interventions improving intestinal motility
Immune Pathway	Elevated pro-inflammatory cytokines (IL-6, TNF-α, etc.); Microglial overactivation	Elevated pro-inflammatory factors → Induce neuroinflammation; Abnormal microglial activation → Disrupted synaptic pruning → Aberrant neural circuit development	(Choi et al., 2016): Maternal IL-17 a elevation induces autistic-like behaviors in offspring mice; (Hsiao et al., 2013): Anti-inflammatory therapy alleviates ASD-like symptoms in mice	Delayed neurodevelopment; Repetitive stereotyped behaviors; Social withdrawal	Anti-IL-6 neutralizing antibodies; Probiotics regulating microglial activity
BBB	Increased intestinal permeability (“leaky gut”); Reduced BBB integrity	Gut dysbiosis → Decreased expression of intestinal tight junction proteins (e.g., occludin) → Toxins/pro-inflammatory substances enter blood; BBB disruption → Neurotoxic substances invade the central nervous system	(Braniste et al., 2014): Microbial metabolites (e.g., p-cresol) increase BBB permeability; (Kelly et al., 2015): Intestinal inflammation exacerbates BBB damage	Systemic inflammation; Accumulation of neurotoxic substances; Central nervous system dysfunction	Prebiotics repairing intestinal barrier; Drugs targeting tight junction proteins

### 4.2 Neuroanatomical pathways of the gut-brain axis

The vagus nerve serves as a critical link between gut microbiota and the brain, communicating with the brain through multiple synaptic connections in the nucleus tractus solitarius of the brainstem (**Table 3**). The ENS, richly distributed in the intestinal wall, forms an independent peripheral nervous system that can transmit information related to digestion, absorption, and immunity upward to the thalamus of the central nervous system. The brain can influence the community structure and function of gut microbiota through the autonomic nervous system by regulating local intestinal motility, transportation, secretion, intestinal permeability, and potentially through luminal secretion of hormones that directly regulate microbial gene expression (Martin et al., 2018). Growing evidence confirms that the vagus nerve system plays a key role in regulating the gut-brain axis (Vuong and Hsiao, 2017; Yu et al., 2020); dysregulation of the vagus nerve system can lead to ASD (Vuong and Hsiao, 2017; Sgritta et al., 2019), while the vagus nerve and ENS can also be directly influenced by neurotransmitter molecules produced by the microbiota (Silva et al., 2020). Abnormalities in 5-HT and kynurenine metabolites in ASD patients affect the activity of intestinal vagal afferents, potentially directly impairing vagal nerve signaling to the brain (Slattery et al., 2006). Golubeva, A V et al (Golubeva et al., 2017)found in animal experiments that ASD model mice (BTBR strain) exhibit significantly delayed overall intestinal transit, accompanied by colonic elongation and reduced fecal water content. Kim, J W et al (Kim et al., 2013). also reported impaired small intestinal peristalsis in an ASD rat model in animal experiments. Healthy gastrointestinal peristalsis largely depends on ENS, and delayed colonic transit in ASD model mice may be related to abnormal development of ENS motor circuits (Golubeva et al., 2017).

### 4.3 Immune pathways

There is a link between ASD and systemic immune dysfunction (excessive inflammation) (**Table 3**) (Zhang et al., 2023). Studies have shown prominent central and peripheral inflammatory responses in ASD patients, with not only systemic immune dysregulation but also abnormal neuroimmune function (Onore et al., 2012). ASD patients often exhibit a state of chronic neuroinflammation, characterized by elevated levels of pro-inflammatory cytokines and chemokines (e.g., TNF-α, IL-6, Interleukin-8(IL-8)) in cerebrospinal fluid (CSF) and activation of microglia in the brain tissue of ASD patients (Vargas et al., 2005; Li et al., 2009; Onore et al., 2012; Zheng et al., 2021). Studies have also confirmed that upregulated expression of IL-6 and Interleukin-17(IL-17) in ASD individuals or animal models induces autistic-like behaviors in the models (Kim et al., 2017; Azhari et al., 2019), and blocking these cytokines with neutralizing antibodies can alleviate autistic behaviors (Choi et al., 2016; Azhari et al., 2019). Microglia, which play a role in innate immune function (Boche et al., 2013), are crucial for neuronal development; they participate in synaptogenesis and are responsible for pruning excess synaptic connections during childhood, a process essential for the development of functional neural circuits (Paolicelli et al., 2011). Elevated levels of pro-inflammatory cytokines and chemokines, along with abnormal activation of microglia in ASD patients, disrupt synaptic maintenance and lead to dysregulation of synaptic pruning, thereby contributing to the occurrence and progression of ASD (Koyama and Ikegaya, 2015). Inflammatory processes are involved in the pathogenesis of ASD; immune activation induces the kynurenine pathway (KP), i.e., the tryptophan catabolic pathway (Yildirim et al., 2023). There is a close interaction between the cytokine IL-6 and KP (Butler et al., 2022; Francis et al., 2022). Yildirim, V et al. confirmed elevated levels of IL-6 and kynurenic acid in ASD patients, suggesting abnormal immune responses in the brains of ASD patients that may lead to abnormal neurotransmission (Yildirim et al., 2023). SHANK3 is a synaptic scaffold protein, and mutations in SHANK3 are associated with ASD. Zhang, L et al (Zhang et al., 2023). demonstrated that vagal SHANK3 can limit excessive inflammation induced by lipopolysaccharide (LPS) in experimental animals, providing new molecular insights into inflammatory dysregulation in some ASD individuals.

### 4.4 Intestinal barrier and blood-brain barrier

The intestinal barrier and BBB are two natural barriers within the gut-brain axis (**Table 3**). Gut microbiota and their related metabolites are involved in regulating the functions of these two barriers (Martin et al., 2018); they can alter the expression of intestinal tight junction proteins (Braniste et al., 2014), influence the growth and development of neuroglial cells, and regulate neuronal activity by modulating the neuro-glial microenvironment. Gut bacteria and their metabolites can regulate intestinal barrier function (Kelly et al., 2015), while the colon of ASD patients may have poor resilience to metabolic stressors such as intestinal inflammation or hypoxia (Golubeva et al., 2017). Thus, under stress or physicochemical insults, the permeability of the BBB may increase, or even intestinal mucosal barrier damage may occur, followed by the release of inflammatory factors. These inflammatory factors can ascend to invade the brain, causing brain function impairment, and invade the gastrointestinal system, leading to gastrointestinal symptoms. Dysbiosis of gut microbiota in ASD patients is also commonly associated with intestinal mucosal barrier damage; such dysbiosis increases intestinal permeability to neurotoxic compounds, resulting in dysregulation of neuromodulatory mechanisms and impairment of normal brain development (De Angelis et al., 2015).

ASD is associated with significantly reduced BBB integrity (Obrenovich, 2018), leading to increased permeability (Fiorentino et al., 2016). The microbiota is involved in regulating BBB integrity. Alterations in levels of gut microbiota-derived metabolites such as p-cresol and its metabolite p-cresyl sulfate in ASD patients affect brain function; these metabolites, known as uremic toxins (Pascucci et al., 2020), can influence BBB permeability, impairing its integrity. Impaired BBB integrity may facilitate the entry of microbiota-derived metabolites into the central nervous system, contributing to the pathogenesis of ASD. Furthermore, systemic immune activation commonly observed in ASD patients may cause destructive changes in the blood-brain barrier (Martin et al., 2018) (**Table 3**).

## 5 Gut microbiota-targeted therapies for ASD

**Figure 2:**
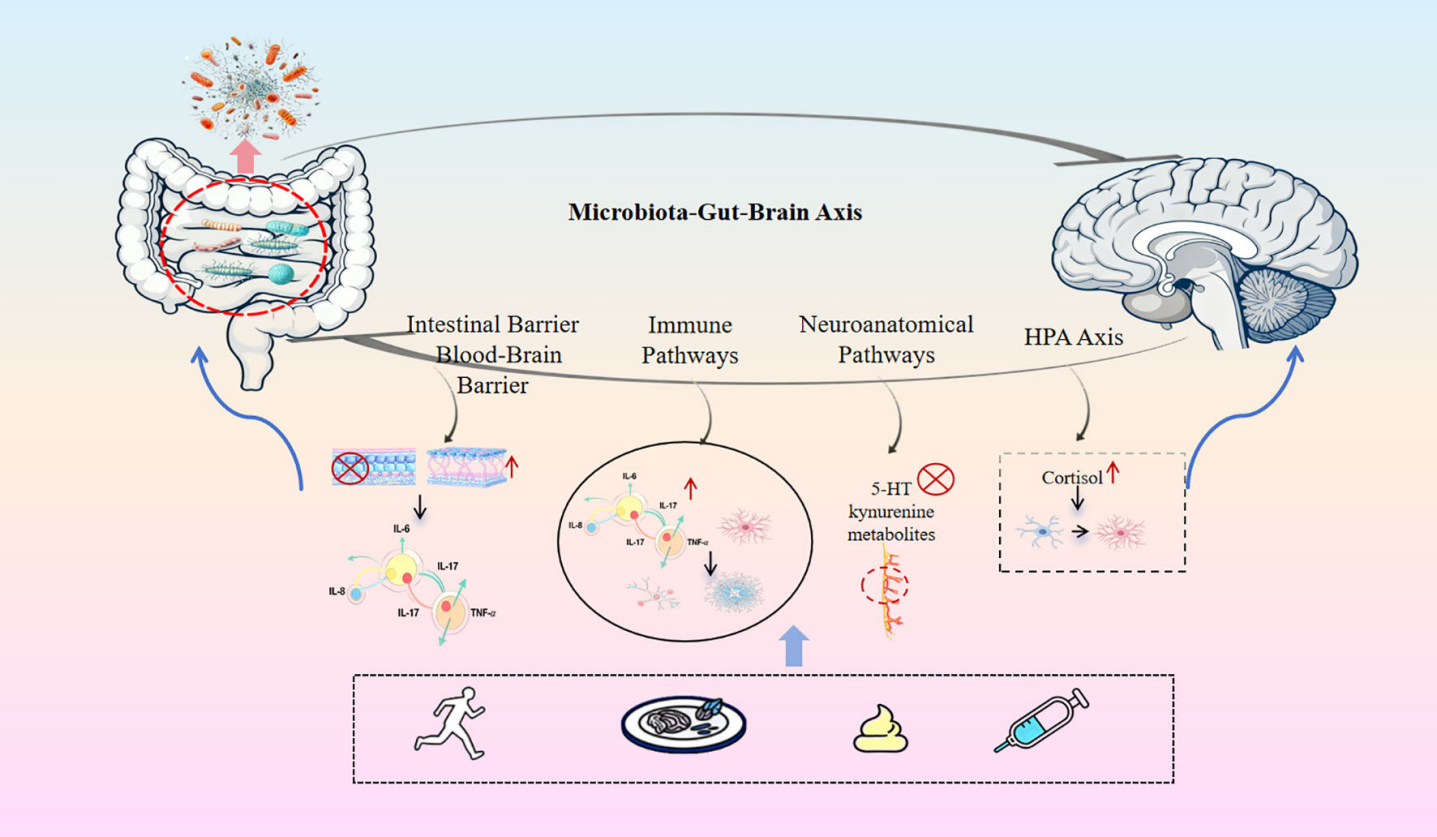
The gut-brain axis in autism spectrum disorder (ASD): mechanisms and interventions. This schematic illustrates the key pathways through which gut microbiome dysbiosis may contribute to the pathophysiology of ASD.The central model depicts four core mechanistic pathways linking the gut and the brain:The Neuroendocrine (HPA Axis) Pathway: Stress-induced cortisol release activates microglia in the brain. The Neural Pathway: Dysregulation of 5-HT and kynurenine metabolite signaling impairs vagus nerve communication.The Immune Pathway: Elevated pro-inflammatory cytokines (e.g., IL-6, IL-17, Tumor Necrosis Factor-alpha(TNF-α)) promote microglial activation and disrupt synaptic pruning.The Barrier Integrity Pathway: Compromised intestinal mucosal integrity and increased BBB permeability facilitate systemic inflammatory responses.The lower panel summarizes four potential microbiota-targeted intervention strategies for modulating the gut-brain axis, including exercise, dietary modification, FMT, and probiotic supplementation.

Currently, drugs and methods for treating ASD are limited, imposing a heavy burden on patient families and society. With the continuous research on the gut microbiota-gut-brain axis, scholars have found that improving gut microbiota can simultaneously alleviate gastrointestinal symptoms and behavioral abnormalities in ASD patients (Li et al., 2021). Therefore, the gut microbiome has gradually become an important target for ASD treatment (Vuong and Hsiao, 2017), and restoring gut microbiota balance has been recognized as an emerging therapy for ASD, including exercise intervention, probiotics, fecal microbiota transplantation, dietary intervention, and antibiotics (**Figure 2**) (**Table 4**).

**Table 4:** Gut microbiota-targeted interventions for ASD.

Intervention type	Specific interventions	Core mechanisms of action	Clinical evidence (human studies)	Experimental evidence (animal/cell studies)	Advantages	Limitations	Potential application prospects
Exercise Therapy	Aerobic exercise (swimming, running); Cognitively engaging activities (martial arts, movement games)	Modulates gut microbiota diversity → Promotes beneficial bacteria proliferation; Induces anti-inflammatory cytokine release → Reduces neuroinflammation; Activates cerebellum-dorsolateral prefrontal cortex co-activation → Improves cognitive function	(Toscano et al., 2018): 48-week exercise intervention improves metabolic markers and quality of life in ASD patients; (Liang et al., 2022): Martial arts enhance executive function and cognitive flexibility	No direct animal experiments, but microbiota-modulated mice models show reduced anxiety after exercise	Safe with no side effects; Easy to implement; Improves overall health	Efficacy depends on adherence; Large individual response variation; Mechanisms not fully clarified	Integration into daily rehabilitation as adjuvant intervention
Dietary Intervention	GFCF;KD;ω-3 supplementation	Reduces pro-inflammatory food intake → Lowers intestinal inflammation; Regulates microbial metabolites (e.g., SCFAs) → Improves gut-brain axis signaling; Optimizes nutritional structure → Supports neurodevelopment	(Whiteley et al., 2010): GFCF diet improves behavior scores in ASD children; Mazahery et al.: ω-3 supplementation reduces IL-6 levels and alleviates inflammation	ASD-like behaviors are reduced in high-sugar diet-induced ASD model mice after KD intervention	Easy to operate; Implementable at home; High safety	Requires strict adherence; Limited long-term efficacy data; Some diets may cause nutritional imbalance	Personalized diet plans combined with microbiota testing
FMT	Healthy donor microbiota transplantation; Standardized microbial preparations	Restores gut microecological balance → Reduces neurotoxic metabolites (e.g., p-cresol); Repairs intestinal barrier → Lowers intestinal permeability; Regulates immune pathways → Reduces systemic inflammation	(Kang et al., 2019): Gastrointestinal and core ASD symptoms improve after FMT, with benefits sustained for 2 years; Sharon et al.: FMT reduces intestinal p-cresol levels	GF mice transplanted with ASD patient microbiota exhibit autistic-like behaviors, which can be reversed by healthy microbiota transplantation	Significant efficacy; Long-term benefits; Regulates microbiota at the root	Difficulty in donor standardization; Infection risks; Insufficient long-term safety data	Precision donor matching combined with metagenomic testing
Prebiotics	GOS;Partially hydrolyzed guar gum	Selectively promotes growth of beneficial bacteria (e.g., Bifidobacterium) → Modulates microbiota structure; Enhances intestinal barrier function → Reduces toxin absorption; Improves intestinal motility → Relieves constipation	(Grimaldi et al., 2018): 6-week GOS intervention reduces antisocial behaviors and improves social scores in ASD children; (Inoue et al., 2019): Partially hydrolyzed guar gum alleviates irritability	Prebiotic-intervened mouse models show increased gut microbiota diversity and reduced anxiety-like behaviors	High safety; Long-term usability; Good tolerance	Slow onset; Efficacy depends on baseline microbiota; Large individual response variation	Combined use with probiotics to enhance efficacy
Probiotics	Bifidobacterium strains; Bifidobacterium longum + fructooligosaccharides	Regulates neurotransmitter (5-HT, DA) metabolism → Improves gut-brain axis signaling; Inhibits pro-inflammatory factor release → Reduces neuroinflammation; Repairs intestinal barrier → Lowers permeability	(Wang et al., 2020): Probiotics + fructooligosaccharides intervention increases Bifidobacterium levels and alleviates gastrointestinal and ASD symptoms; (Shaaban et al., 2018): Probiotics improve behavioral and intestinal symptoms	Social ability is enhanced and gut microbiota imbalance is improved in ASD model mice supplemented with Bifidobacterium	High safety; Easily accessible; Long-term administrable	Strain-specific efficacy; Efficacy affected by dosage and duration; Intolerance in some patients	Personalized probiotic formulations based on microbiota characteristics

### 5.1 Exercise therapy

In recent years, attention has been paid to the impact of exercise on the microbiota. Exercise can balance the relationship between beneficial and pathogenic bacteria in the gut (Xue et al., 2023), and exercise intervention can improve motor skills and behavioral outcomes in autistic children (Yu et al., 2018), including stereotyped behaviors, social-emotional functions, cognition, and attention (Bremer et al., 2016; Toscano et al., 2018). Toscano, CVA et al. conducted a 48-week exercise intervention in ASD patients and found that exercise therapy had beneficial effects on metabolic indicators and quality of life in ASD patients (Toscano et al., 2018). Liang, X et al. concluded in their research analysis that chronic exercise intervention has a small to moderate significant impact on overall executive functions (EFs), inhibitory control, and cognitive flexibility in children and adolescents with ASD. Cognitively engaging exercises (e.g., martial arts and movement games) have beneficial effects on overall EFs in children and adolescents with ASD. This may be because exercise promotes co-activation between the cerebellum and dorsolateral prefrontal cortex in ASD patients, thereby improving their cognitive function (Liang et al., 2022). Cytokines released after exercise can play an important role in regulating neuronal metabolism (Gabriele et al., 2014). Although exercise therapy can improve symptoms in ASD patients (Schmitz et al., 2017), there are still few studies on how exercise therapy exerts therapeutic effects by improving the gut microbiota of ASD patients.

### 5.2 Dietary intervention

Food preferences and dietary patterns are considered key factors influencing the development of autism spectrum disorder. Moreover, maternal diet is also related to the development of ASD in offspring (Sullivan et al., 2014). Adequate intake of folic acid and vitamins during pregnancy can reduce the risk of ASD (Suren et al., 2013), while a high-fat diet during pregnancy increases the risk of ASD (Peretti et al., 2019). Therefore, dietary intervention is often used as one of the means to treat ASD. Many specialized diets are considered beneficial for ASD. For example, a gluten-free diet (GFD) is thought to improve ASD-related behavioral and intellectual problems (Croall et al., 2021), and a gluten-free casein-free diet (GFCF) is also believed to have a positive impact on the health status of ASD patients (Whiteley et al., 2010). A high-fat (65-90%) ketogenic diet (KD) is often used to treat ASD, and omega-3 fatty acids (ω-3), commonly used as food supplements, are also regarded as effective complementary and alternative therapeutic agents for ASD (Bent et al., 2009). ASD can lead to increased inflammatory cytokines, oxidative stress, and neurotransmitter dysfunction, so autistic children often have omega-3 long-chain polyunsaturated fatty acid (PUFA) metabolic defects (Das, 2013). Supplementation with ω-3 is beneficial to brain and visual development, function, as well as behavior and emotional regulation (Kidd, 2007). Fecal amino acids can also be used to design personalized diets to prevent or minimize ASD-related cognitive impairments. The amino acid profile from protein sources can be applied in the early stages of autism development to prevent or minimize cognitive impairments and neurodevelopmental dysfunctions associated with the disease (Chamtouri et al., 2023b).

### 5.3 Fecal microbiota transplantation

Fecal microbiota transplantation (FMT) is a safe and effective method for intestinal microecological reconstruction (Ianiro et al., 2018). As a potential therapy for gastrointestinal (GI) problems in ASD children, it has attracted increasing attention. FMT is the most effective treatment for recurrent Clostridium difficile infection (Bagdasarian et al., 2015). Microbiota transfer therapy aims to partially improve autistic behaviors by reducing the absorption of neurotoxic compounds from environmental sources or specific intestinal bacterial strains (Dolen, 2015; Pascucci et al., 2020). Animal studies have shown that transplanting fecal microbiota from patients with neurological diseases can cause GF mice to exhibit typical disease symptoms (Davies et al., 2021). Kang, D W et al. confirmed in clinical studies that ASD patients receiving FMT treatment showed significant improvements in gastrointestinal symptoms, autism-related symptoms, and gut microbiota, with long-term benefits; symptoms remained improved 2 years after treatment (Kang et al., 2019). Further clinical studies found that FMT can sustainably reduce the excessively high p-cresol levels in the intestines of autistic children and improve intestinal symptoms, emotional and behavioral abnormalities in ASD patients (Li et al., 2021).

### 5.4 Prebiotics

The International Scientific Association for Probiotics and Prebiotics (ISAPP) defines prebiotics as “substances selectively utilized by host microorganisms to confer a health benefit” (Swanson et al., 2020). Both prebiotics and probiotics can help restore the gut microbiota to normal levels and exert effects through the gut-brain axis, influencing neurotransmission and psychological status (Wang et al., 2020). Prebiotics commonly used in clinical treatment of ASD mainly include carrot powder, vitamin A, partially hydrolyzed guar gum, and galactooligosaccharides. Grimaldi, R et al. showed in a 6-week experiment that supplementation with galactooligosaccharide (GOS) prebiotics can reduce antisocial behaviors in autistic children and improve their scores on social skills scales (Grimaldi et al., 2018). Inoue, R also confirmed the therapeutic effect of prebiotics on ASD patients in his research; consumption of partially hydrolyzed guar gum prebiotics can reduce irritable behaviors in autistic children (Inoue et al., 2019).

### 5.5 Probiotics

Specifically, clinical and preclinical studies have demonstrated that certain probiotic strains, such as Lactobacillus and Bifidobacterium blends, can improve gastrointestinal symptoms and social responsiveness in ASD subjects, potentially through mechanisms involving microbial metabolite regulation and gut barrier integrity (Alli et al., 2022), and consumption of probiotics can improve gut microbiota and benefit health. Studies have shown that probiotics can produce and/or regulate tissue neurotransmitter levels, acting through the gut-brain axis (Dinan et al., 2013), so probiotics are regarded as alternative and complementary supplements for ASD treatment (Cekici and Sanlier, 2019). Relevant studies have confirmed that oral probiotics can reduce baseline or induced anxiety-like behaviors (Bravo et al., 2011; Messaoudi et al., 2011), weaken induced compulsive-like behaviors (Kantak et al., 2014), improve inflammation-related disease behaviors, and even normalize the developmental trajectory of emotion-related behaviors after early-life stress (D'Mello et al., 2015). Ying Wang et al. found in clinical studies that the levels of Bifidobacterium and Bifidobacterium longum in ASD patients were lower than those in the control group. After intervention with probiotics + fructooligosaccharides, the levels of Bifidobacterium and Bifidobacterium longum increased, gastrointestinal symptoms and ASD-related symptoms were alleviated, and the status of excessive serotonin and DA metabolic disorders in ASD patients was also reduced (Wang et al., 2020). Sanaa Y. Shaaban et al. confirmed in their research that probiotics can improve both behavioral and gastrointestinal symptoms of autism spectrum disorder (Shaaban et al., 2018). El-Ansary, A et al. confirmed in their research that probiotics stimulate inhibitory neurotransmission and can reverse impaired social interaction related to ASD (El-Ansary et al., 2018).

Although it has been well established that intervention strategies targeting the gut microbiota—such as exercise therapy, dietary intervention, FMT, prebiotics, and probiotics—are effective in the management of ASD, these approaches are associated with several limitations. Exercise therapy is advantageous due to its safety profile, minimal side effects, and ease of implementation. However, its underlying mechanisms remain poorly understood, and treatment outcomes are highly dependent on patient adherence. Furthermore, individual responses vary significantly, and therapeutic effects are often inconsistent, making it more suitable for integration into routine rehabilitation programs as an adjunctive measure in clinical practice. Dietary interventions are generally safe, practical, and can be administered at home. Nevertheless, they require strict adherence, may lead to nutritional imbalances, and lack sufficient long-term efficacy data. The development of personalized dietary plans guided by microbiota profiling may enhance their therapeutic potential. FMT demonstrates notable clinical efficacy, offers sustained benefits, and enables fundamental modulation of the gut microbiota. However, challenges include donor standardization, risk of infection, and insufficient long-term safety evidence. The integration of metagenomic analysis for precise donor-recipient matching could significantly improve its clinical applicability. Both prebiotics and probiotics exhibit favorable safety profiles, tolerability, and suitability for prolonged use. Yet, the efficacy of probiotics is strain-specific and influenced by dosage, duration of treatment, and individual tolerance, with some patients experiencing adverse reactions. Prebiotics, on the other hand, act gradually, and their effectiveness is contingent upon baseline microbial composition and exhibits considerable inter-individual variability. In clinical settings, combining personalized probiotic formulations tailored to individual microbiota profiles with prebiotic supplementation may yield superior therapeutic outcomes.

## 6 Conclusion

Current research has conducted multi-dimensional explorations on the association between the gut microbiota-gut-brain axis and ASD, confirming that gut microbiota, as a key environmental factor, participates in the occurrence and development of ASD through multiple pathways such as immunity, neuroendocrinology, neurotransmitters, and metabolism. Studies have found that ASD patients exhibit gut microbiota dysbiosis, and abnormal levels of their metabolites—such as short-chain fatty acids, phenolic compounds, bile acids, and amino acids—can exacerbate gastrointestinal symptoms and behavioral abnormalities in ASD patients by affecting processes like BBB permeability, neuroinflammatory responses, synaptic pruning, and neurotransmitter balance. Based on this, gut microbiota-targeted intervention strategies have become a research focus: exercise therapy can improve cognitive and behavioral functions in ASD patients by regulating microbiota balance; dietary interventions (such as GFD, ketogenic diets, and omega-3 fatty acid supplementation) can alleviate symptoms by optimizing nutritional structure and microbiota metabolic environment; FMT achieves long-term improvement of gastrointestinal and behavioral symptoms by reconstructing intestinal microecology; and prebiotic and probiotic interventions exert therapeutic effects by regulating microbiota metabolites, repairing intestinal barriers, and influencing neurotransmission.

However, existing research still has many limitations: the specific molecular mechanisms by which microbiota metabolites regulate neural functions have not been fully elucidated, such as the details of how SCFAs affect neuroplasticity through GPR41 receptor or HDAC inhibition remaining to be explored; the contradiction between standardization and individualization in clinical interventions is prominent, with significant differences in response to microbiota interventions among different ASD subtypes, and a lack of precise intervention plans based on multi-omics characteristics; research on early intervention is insufficient, and the mechanism by which microbiota regulation during the critical window of neurodevelopment (such as pregnancy and infancy) affects ASD prevention has not yet formed a systematic understanding.

Future studies should focus on verifying the causal relationship of the “microbiota-metabolite-neural pathway,” clarify core regulatory targets by combining organoid models, gene editing technologies, and multi-omics integration analysis; build a “microbiota-clinical phenotype” association model relying on artificial intelligence algorithms to develop personalized intervention strategies, promoting the combined application of exercise, diet, FMT, and prebiotics/probiotics; strengthen research on the intergenerational transmission of maternal microbiota and ASD risk during pregnancy, establish an early warning and intervention system, and ultimately achieve a breakthrough from mechanism research to clinical translation, providing new ideas and scientific basis for the precise prevention and treatment of ASD. In future studies, we believe it is highly meaningful to focus on the following points: (1) the microbiota changes during pregnancy could help the prevention and treatment of ASD; (2) Kynurenic acid, L-Kynurenine, and IL-6 could be novel biomarkers for ASD diagnosis; (3) Multi-omics in combination with AI could build “microbiota-clinical phenotype” for individualized medication.

## Glossary

ASD: Autism Spectrum Disorder
NCBI: National Center for Biotechnology Information
GP: General Practitioner
NT: Neurotypical
TD: Typically Developing
5AV: 5-aminovaleric acid
RBPs: Ribosomal Binding Proteins
RNA: Ribonucleic Acid
GF: Germ-Free
5-HT: 5-Hydroxytryptamine
GABA: Gamma-Aminobutyric Acid
DA: Dopamine
ENS: Enteric Nervous System
IL-6: Interleukin-6
IL-8: Interleukin-8
IL-17: Interleukin-17
TNF-α: Tumor Necrosis Factor-alpha
4EP: 4-Ethylphenol
4EPS: 4-Ethylphenyl sulfate
CGRP: CalcitoninGene-Related Peptide
SCFAs: Short-Chain Fatty Acids
GPR41: G Protein-Coupled Receptor 41
HDAC: Histone Deacetylase
CNS: Central Nervous System
BBB: Blood-Brain Barrier
PPA: Propionic Acid
HPA: Hypothalamic-Pituitary-Adrenal
CSF: Cerebrospinal Fluid
KP: Kynurenine Pathway
LPS: Lipopolysaccharide
EFs: Executive Functions
GFD: Gluten-Free Diet
GFCF: Gluten-Free Casein-Free diet
KD: Ketogenic Diet
ω-3: Omega-3 Fatty Acids
PUFA: Polyunsaturated Fatty Acid
FMT: Fecal Microbiota Transplantation
GI: Gastrointestinal
ISAPP: International Scientific Association for Probiotics and Prebiotics
GOS: Galactooligosaccharide

## References

[B1] Alemi F. Poole D. P. Chiu J. Schoonjans K. Cattaruzza F. Grider J. . (2013). The receptor TGR5 mediates the prokinetic actions of intestinal bile acids and is required for normal defecation in mice. Gastroenterology 144, 145–154. doi: 10.1053/j.gastro.2012.09.055, PMID: 23041323 PMC6054127

[B2] Alharthi A. Alhazmi S. Alburae N. Bahieldin A. (2022). The human gut microbiome as a potential factor in autism spectrum disorder. Int. J. Mol. Sci. 23, 1363. doi: 10.3390/ijms23031363, PMID: 35163286 PMC8835713

[B3] Al-Lahham S. H. Peppelenbosch M. P. Roelofsen H. Vonk R. J. Venema K. (2010). Biological effects of propionic acid in humans; metabolism, potential applications and underlying mechanisms. Biochim. Biophys. Acta 1801, 1175–1183. doi: 10.1016/j.bbalip.2010.07.007, PMID: 20691280

[B4] Alli S. R. Gorbovskaya I. Liu J. C. W. Kolla N. J. Brown L. Muller D. J. (2022). The gut microbiome in depression and potential benefit of prebiotics, probiotics and synbiotics: A systematic review of clinical trials and observational studies. Int. J. Mol. Sci. 23, 4494. doi: 10.3390/ijms23094494, PMID: 35562885 PMC9101152

[B5] Al-Otaish H. Al-Ayadhi L. Bjorklund G. Chirumbolo S. Urbina M. A. El-Ansary A. (2018). Relationship between absolute and relative ratios of glutamate, glutamine and GABA and severity of autism spectrum disorder. Metab. Brain Dis. 33, 843–854. doi: 10.1007/s11011-018-0186-6, PMID: 29397522

[B6] Al-Owain M. Kaya N. Al-Shamrani H. Al-Bakheet A. Qari A. Al-Muaigl S. . (2013). Autism spectrum disorder in a child with propionic acidemia. JIMD Rep. 7, 63–66. doi: 10.1007/8904_2012_143, PMID: 23430497 PMC3573175

[B7] Ames J. L. Windham G. C. Lyall K. Pearl M. Kharrazi M. Yoshida C. K. . (2020). Neonatal thyroid stimulating hormone and subsequent diagnosis of autism spectrum disorders and intellectual disability. Autism Res. 13, 444–455. doi: 10.1002/aur.2247, PMID: 31823519

[B8] Azhari A. Azizan F. Esposito G. (2019). A systematic review of gut-immune-brain mechanisms in Autism Spectrum Disorder. Dev. Psychobiol 61, 752–771. doi: 10.1002/dev.21803, PMID: 30523646

[B9] Bagdasarian N. Rao K. Malani P. N. (2015). Diagnosis and treatment of Clostridium difficile in adults: a systematic review. JAMA 313, 398–408. doi: 10.1001/jama.2014.17103, PMID: 25626036 PMC6561347

[B10] Bent S. Bertoglio K. Hendren R. L. (2009). Omega-3 fatty acids for autistic spectrum disorder: a systematic review. J. Autism Dev. Disord. 39, 1145–1154. doi: 10.1007/s10803-009-0724-5, PMID: 19333748 PMC2710498

[B11] Boche D. Perry V. H. Nicoll J. A. (2013). Review: activation patterns of microglia and their identification in the human brain. Neuropathol. Appl. Neurobiol. 39, 3–18. doi: 10.1111/nan.12011, PMID: 23252647

[B12] Borodovitsyna O. Flamini M. D. Chandler D. J. (2018). Acute stress persistently alters locus coeruleus function and anxiety-like behavior in adolescent rats. Neuroscience 373, 7–19. doi: 10.1016/j.neuroscience.2018.01.020, PMID: 29341884

[B13] Braniste V. Al-Asmakh M. Kowal C. Anuar F. Abbaspour A. Toth M. . (2014). The gut microbiota influences blood-brain barrier permeability in mice. Sci. Transl. Med. 6, 263ra158. doi: 10.1126/scitranslmed.3009759, PMID: 25411471 PMC4396848

[B14] Bravo J. A. Forsythe P. Chew M. V. Escaravage E. Savignac H. M. Dinan T. G. . (2011). Ingestion of Lactobacillus strain regulates emotional behavior and central GABA receptor expression in a mouse via the vagus nerve. Proc. Natl. Acad. Sci. U.S.A. 108, 16050–16055. doi: 10.1073/pnas.1102999108, PMID: 21876150 PMC3179073

[B15] Bremer E. Crozier M. Lloyd M. (2016). A systematic review of the behavioural outcomes following exercise interventions for children and youth with autism spectrum disorder. Autism 20, 899–915. doi: 10.1177/1362361315616002, PMID: 26823546

[B16] Butler M. I. Long-Smith C. Moloney G. M. Morkl S. O'Mahony S. M. Cryan J. F. . (2022). The immune-kynurenine pathway in social anxiety disorder. Brain Behav. Immun. 99, 317–326. doi: 10.1016/j.bbi.2021.10.020, PMID: 34758380

[B17] Cekici H. Sanlier N. (2019). Current nutritional approaches in managing autism spectrum disorder: A review. Nutr. Neurosci. 22, 145–155. doi: 10.1080/1028415X.2017.1358481, PMID: 28762296

[B18] Chamtouri M. Gaddour N. Merghni A. Mastouri M. Arboleya S. de Los Reyes-Gavilán C. G. (2023a). Age and severity-dependent gut microbiota alterations in Tunisian children with autism spectrum disorder. Sci. Rep. 13, 18218–18218. doi: 10.1038/s41598-023-45534-0, PMID: 37880312 PMC10600251

[B19] Chamtouri M. Merghni A. Salazar N. Redruello B. Gaddour N. Mastouri M. . (2023b). An overview on fecal profiles of amino acids and related amino-derived compounds in children with autism spectrum disorder in Tunisia. Molecules 28, 3269. doi: 10.3390/molecules28073269, PMID: 37050030 PMC10096484

[B20] Chang X. Zhang Y. Chen X. Li S. Mei H. Xiao H. . (2024). Gut microbiome and serum amino acid metabolome alterations in autism spectrum disorder. Sci. Rep. 14, 4037. doi: 10.1038/s41598-024-54717-2, PMID: 38369656 PMC10874930

[B21] Chatterjee I. Getselter D. Ghanayem N. Harari R. Davis L. Bel S. . (2023). CHD8 regulates gut epithelial cell function and affects autism-related behaviors through the gut-brain axis. Transl. Psychiatry 13, 305. doi: 10.1038/s41398-023-02611-2, PMID: 37783686 PMC10545671

[B22] Chen T. Kim C. Y. Kaur A. Lamothe L. Shaikh M. Keshavarzian A. . (2017). Dietary fibre-based SCFA mixtures promote both protection and repair of intestinal epithelial barrier function in a Caco-2 cell model. Food Funct. 8, 1166–1173. doi: 10.1039/C6FO01532H, PMID: 28174773

[B23] Chernikova M. A. Flores G. D. Kilroy E. Labus J. S. Mayer E. A. Aziz-Zadeh L. . (2021). The brain-gut-microbiome system: pathways and implications for autism spectrum disorder. Nutrients 13, 4497. doi: 10.3390/nu13124497, PMID: 34960049 PMC8704412

[B24] Choi G. B. Yim Y. S. Wong H. Kim S. Kim H. Kim S. V. . (2016). The maternal interleukin-17a pathway in mice promotes autism-like phenotypes in offspring. Science 351, 933–939. doi: 10.1126/science.aad0314, PMID: 26822608 PMC4782964

[B25] Choi J. . (2018). Pathophysiological and neurobehavioral characteristics of a propionic acid-mediated autism-like rat model. PloS One 13, e0192925. doi: 10.1371/journal.pone.0192925, PMID: 29447237 PMC5814017

[B26] Collado M. C. Rautava S. Aakko J. Isolauri E. Salminen S. (2016). Human gut colonisation may be initiated in *utero* by distinct microbial communities in the placenta and amniotic fluid. Sci. Rep. 6, 23129. doi: 10.1038/srep23129, PMID: 27001291 PMC4802384

[B27] Coretti L. Paparo L. Riccio M. P. Amato F. Cuomo M. Natale A. . (2018). Gut microbiota features in young children with autism spectrum disorders. Front. Microbiol. 9, 3146. doi: 10.3389/fmicb.2018.03146, PMID: 30619212 PMC6305749

[B28] Cornell J. Salinas S. Huang H. Y. Zhou M. (2022). Microglia regulation of synaptic plasticity and learning and memory. Neural Regener. Res. 17, 705–716. doi: 10.4103/1673-5374.322423, PMID: 34472455 PMC8530121

[B29] Croall I. D. Hoggard N. Hadjivassiliou M. (2021). Gluten and autism spectrum disorder. Nutrients 13, 572. doi: 10.3390/nu13020572, PMID: 33572226 PMC7915454

[B30] D'Mello C. Ronaghan N. Zaheer R. Dicay M. Le T. MacNaughton W. K. . (2015). Probiotics improve inflammation-associated sickness behavior by altering communication between the peripheral immune system and the brain. J. Neurosci. 35, 10821–10830. doi: 10.1523/JNEUROSCI.0575-15.2015, PMID: 26224864 PMC6605112

[B31] Damiani F. Cornuti S. Tognini P. (2023). The gut-brain connection: Exploring the influence of the gut microbiota on neuroplasticity and neurodevelopmental disorders. Neuropharmacology 231, 109491. doi: 10.1016/j.neuropharm.2023.109491, PMID: 36924923

[B32] Das U. N. (2013). Autism as a disorder of deficiency of brain-derived neurotrophic factor and altered metabolism of polyunsaturated fatty acids. Nutrition 29, 1175–1185. doi: 10.1016/j.nut.2013.01.012, PMID: 23911220

[B33] Davies C. Mishra D. Eshraghi R. S. Mittal J. Sinha R. Bulut E. . (2021). Altering the gut microbiome to potentially modulate behavioral manifestations in autism spectrum disorders: A systematic review. Neurosci. Biobehav. Rev. 128, 549–557. doi: 10.1016/j.neubiorev.2021.07.001, PMID: 34271306

[B34] Davis E. P. Sandman C. A. (2010). The timing of prenatal exposure to maternal cortisol and psychosocial stress is associated with human infant cognitive development. Child Dev. 81, 131–148. doi: 10.1111/j.1467-8624.2009.01385.x, PMID: 20331658 PMC2846100

[B35] Day F. O'Sullivan J. Pook C. (2023). 4-Ethylphenol-fluxes, metabolism and excretion of a gut microbiome derived neuromodulator implicated in autism. Front. Mol. Biosci. 10, 1267754. doi: 10.3389/fmolb.2023.1267754, PMID: 37900921 PMC10602680

[B36] De Angelis M. Piccolo M. Vannini L. Siragusa S. De Giacomo A. Serrazzanetti D. I. . (2013). Fecal microbiota and metabolome of children with autism and pervasive developmental disorder not otherwise specified. PloS One 8, e76993. doi: 10.1371/journal.pone.0076993, PMID: 24130822 PMC3793965

[B37] De Angelis M. Francavilla R. Piccolo M. De Giacomo A. Gobbetti M. (2015). Autism spectrum disorders and intestinal microbiota. Gut Microbes 6, 207–213. doi: 10.1080/19490976.2015.1035855, PMID: 25835343 PMC4616908

[B38] Deng W. Wang S. Li F. Wang F. Xing Y. P. Li Y. . (2022). Gastrointestinal symptoms have a minor impact on autism spectrum disorder and associations with gut microbiota and short-chain fatty acids. Front. Microbiol. 13, 1000419. doi: 10.3389/fmicb.2022.1000419, PMID: 36274684 PMC9585932

[B39] Dinan T. G. Cryan J. F. (2017). Gut-brain axis in 2016: Brain-gut-microbiota axis - mood, metabolism and behaviour. Nat. Rev. Gastroenterol. Hepatol. 14, 69–70. doi: 10.1038/nrgastro.2016.200, PMID: 28053341

[B40] Dinan T. G. Stanton C. Cryan J. F. (2013). Psychobiotics: a novel class of psychotropic. Biol. Psychiatry 74, 720–726. doi: 10.1016/j.biopsych.2013.05.001, PMID: 23759244

[B41] Dodd D. Spitzer M. H. Van Treuren W. Merrill B. D. Hryckowian A. J. Higginbottom S. K. . (2017). A gut bacterial pathway metabolizes aromatic amino acids into nine circulating metabolites. Nature 551, 648–652. doi: 10.1038/nature24661, PMID: 29168502 PMC5850949

[B42] Dolen G. (2015). Autism: Oxytocin, serotonin, and social reward. Soc. Neurosci. 10, 450–465. doi: 10.1080/17470919.2015.1087875, PMID: 26317636

[B43] El-Ansary A. Bacha A. B. Bjorklund G. Al-Orf N. Bhat R. S. Moubayed N. . (2018). Probiotic treatment reduces the autistic-like excitation/inhibition imbalance in juvenile hamsters induced by orally administered propionic acid and clindamycin. Metab. Brain Dis. 33, 1155–1164. doi: 10.1007/s11011-018-0212-8, PMID: 29582256

[B44] Elsabbagh M. Divan G. Koh Y. J. Kim Y. S. Kauchali S. Marcin C. . (2012). Global prevalence of autism and other pervasive developmental disorders. Autism Res. 5, 160–179. doi: 10.1002/aur.239, PMID: 22495912 PMC3763210

[B45] Erhardt S. Schwieler L. Imbeault S. Engberg G. (2017). The kynurenine pathway in schizophrenia and bipolar disorder. Neuropharmacology 112, 297–306. doi: 10.1016/j.neuropharm.2016.05.020, PMID: 27245499

[B46] Erny D. De Angelis AL. Jaitin D. Wieghofer P. Staszewski O. David E. . (2015). Host microbiota constantly control maturation and function of microglia in the CNS. Nat. Neurosci. 18, 965–977. doi: 10.1038/nn.4030, PMID: 26030851 PMC5528863

[B47] Fairbrass K. M. Lovatt J. Barberio B. Yuan Y. Gracie D. J. Ford A. C. . (2022). Bidirectional brain-gut axis effects influence mood and prognosis in IBD: a systematic review and meta-analysis. Gut 71, 1773–1780. doi: 10.1136/gutjnl-2021-325985, PMID: 34725197

[B48] Falomir-Lockhart L. J. Cavazzutti G. F. Gimenez E. Toscani A. M. (2019). Fatty acid signaling mechanisms in neural cells: fatty acid receptors. Front. Cell Neurosci. 13, 162. doi: 10.3389/fncel.2019.00162, PMID: 31105530 PMC6491900

[B49] Filippova Y. Y. Devyatova E. V. Alekseeva A. S. Burmistrova A. L. (2022). Cytokines and neurotrophic factors in the severity assessment of children autism. Klin Lab. Diagn. 67, 647–651. doi: 10.51620/0869-2084-2022-67-11-647-651, PMID: 36398773

[B50] Fiorentino M. Sapone A. Senger S. Camhi S. S. Kadzielski S. M. Buie T. M. . (2016). Blood-brain barrier and intestinal epithelial barrier alterations in autism spectrum disorders. Mol. Autism 7, 49. doi: 10.1186/s13229-016-0110-z, PMID: 27957319 PMC5129651

[B51] Fischer S. (2021). The hypothalamus in anxiety disorders. Handb. Clin. Neurol. 180, 149–160. doi: 10.1016/B978-0-12-820107-7.00009-4, PMID: 34225926

[B52] Francis H. M. Stevenson R. J. Tan L. S. Y. Ehrenfeld L. Byeon S. Attuquayefio T. . (2022). Kynurenic acid as a biochemical factor underlying the association between Western-style diet and depression: A cross-sectional study. Front. Nutr. 9, 945538. doi: 10.3389/fnut.2022.945538, PMID: 36299996 PMC9589270

[B53] Fulceri F. Morelli M. Santocchi E. Cena H. Del Bianco T. Narzisi A. . (2016). Gastrointestinal symptoms and behavioral problems in preschoolers with Autism Spectrum Disorder. Dig Liver Dis. 48, 248–254. doi: 10.1016/j.dld.2015.11.026, PMID: 26748423

[B54] Gabriele S. Sacco R. Altieri L. Neri C. Urbani A. Bravaccio C. . (2016). Slow intestinal transit contributes to elevate urinary p-cresol level in Italian autistic children. Autism Res. 9, 752–759. doi: 10.1002/aur.1571, PMID: 26437875

[B55] Gabriele S. Sacco R. Persico A. M. (2014). Blood serotonin levels in autism spectrum disorder: a systematic review and meta-analysis. Eur. Neuropsychopharmacol. 24, 919–929. doi: 10.1016/j.euroneuro.2014.02.004, PMID: 24613076

[B56] Gadaleta R. M. van Erpecum K. J. Oldenburg B. Willemsen E. C. Renooij W. Murzilli S. . (2011). Farnesoid X receptor activation inhibits inflammation and preserves the intestinal barrier in inflammatory bowel disease. Gut 60, 463–472. doi: 10.1136/gut.2010.212159, PMID: 21242261

[B57] Gagliano H. Delgado-Morales R. Sanz-Garcia A. Armario A. (2014). High doses of the histone deacetylase inhibitor sodium butyrate trigger a stress-like response. Neuropharmacology 79, 75–82. doi: 10.1016/j.neuropharm.2013.10.031, PMID: 24212060

[B58] Gao J. Zou J. Yang L. Zhao J. Wang L. Liu T. . (2022). Alteration of peripheral cortisol and autism spectrum disorder: A meta-analysis. Front. Psychiatry 13, 928188. doi: 10.3389/fpsyt.2022.928188, PMID: 35911217 PMC9334910

[B59] Gevi F. Belardo A. Zolla L. (2020). A metabolomics approach to investigate urine levels of neurotransmitters and related metabolites in autistic children. Biochim. Biophys. Acta Mol. Basis Dis. 1866, 165859. doi: 10.1016/j.bbadis.2020.165859, PMID: 32512190

[B60] Golubeva A. V. Joyce S. A. Moloney G. Burokas A. Sherwin E. Arboleya S. . (2017). Microbiota-related changes in bile acid & Tryptophan metabolism are associated with gastrointestinal dysfunction in a mouse model of autism. EBioMedicine 24, 166–178. doi: 10.1016/j.ebiom.2017.09.020, PMID: 28965876 PMC5652137

[B61] Goode T. D. Ressler R. L. Acca G. M. Miles O. W. Maren S. (2019). Bed nucleus of the stria terminalis regulates fear to unpredictable threat signals. Elife 8, e46525. doi: 10.7554/eLife.46525.027, PMID: 30946011 PMC6456295

[B62] Grimaldi R. Gibson G. R. Vulevic J. Giallourou N. Castro-Mejia J. L. Hansen L. H. . (2018). A prebiotic intervention study in children with autism spectrum disorders (ASDs). Microbiome 6, 133. doi: 10.1186/s40168-018-0523-3, PMID: 30071894 PMC6091020

[B63] Guo X. Okpara E. S. Hu W. Yan C. Wang Y. Liang Q. . (2022). Interactive relationships between intestinal flora and bile acids. Int. J. Mol. Sci. 23, 8343. doi: 10.3390/ijms23158343, PMID: 35955473 PMC9368770

[B64] Guo C. Huo Y. J. Li Y. Han Y. Zhou D. (2022). Gut-brain axis: Focus on gut metabolites short-chain fatty acids. World J. Clin. cases 10, 1754–1763. doi: 10.12998/wjcc.v10.i6.1754, PMID: 35317140 PMC8891794

[B65] Heredia D. J. Gershon M. D. Koh S. D. Corrigan R. D. Okamoto T. Smith T. K. . (2013). Important role of mucosal serotonin in colonic propulsion and peristaltic reflexes: *in vitro* analyses in mice lacking tryptophan hydroxylase 1. J. Physiol. 591, 5939–5957. doi: 10.1113/jphysiol.2013.256230, PMID: 24127620 PMC3872763

[B66] Hirota T. King B. H. (2023). Autism spectrum disorder: A review. JAMA 329, 157–168. doi: 10.1001/jama.2022.23661, PMID: 36625807

[B67] Hrnciarova J. Kubelkova K. Bostik V. Rychlik I. Karasova D. Babak V. . (2024). Modulation of gut microbiome and autism symptoms of ASD children supplemented with biological response modifier: A randomized, double-blinded, placebo-controlled pilot study. Nutrients 16, 1988. doi: 10.3390/nu16131988, PMID: 38999736 PMC11243103

[B68] Hsiao E. Y. McBride S. W. Hsien S. Sharon G. Hyde E. R. McCue T. . (2013). Microbiota modulate behavioral and physiological abnormalities associated with neurodevelopmental disorders. Cell 155, 1451–1463. doi: 10.1016/j.cell.2013.11.024, PMID: 24315484 PMC3897394

[B69] Ianiro G. Masucci L. Quaranta G. Simonelli C. Lopetuso L. R. Sanguinetti M. . (2018). Randomised clinical trial: faecal microbiota transplantation by colonoscopy plus vancomycin for the treatment of severe refractory Clostridium difficile infection-single versus multiple infusions. Aliment Pharmacol. Ther. 48, 152–159. doi: 10.1111/apt.14816, PMID: 29851107

[B70] Iglesias-Vazquez L. Van Ginkel R. G. Arija V. Canals J. (2020). Composition of gut microbiota in children with autism spectrum disorder: A systematic review and meta-analysis. Nutrients 12, 792. doi: 10.3390/nu12030792, PMID: 32192218 PMC7146354

[B71] Inagaki T. Moschetta A. Lee Y. K. Peng L. Zhao G. Downes M. . (2006). Regulation of antibacterial defense in the small intestine by the nuclear bile acid receptor. Proc. Natl. Acad. Sci. U.S.A. 103, 3920–3925. doi: 10.1073/pnas.0509592103, PMID: 16473946 PMC1450165

[B72] Inoue R. Sakaue Y. Kawada Y. Tamaki R. Yasukawa Z. Ozeki M. . (2019). Dietary supplementation with partially hydrolyzed guar gum helps improve constipation and gut dysbiosis symptoms and behavioral irritability in children with autism spectrum disorder. J. Clin. Biochem. Nutr. 64, 217–223. doi: 10.3164/jcbn.18-105, PMID: 31138955 PMC6529696

[B73] Iovene M. R. Bombace F. Maresca R. Sapone A. Iardino P. Picardi A. . (2017). Intestinal dysbiosis and yeast isolation in stool of subjects with autism spectrum disorders. Mycopathologia 182, 349–363. doi: 10.1007/s11046-016-0068-6, PMID: 27655151

[B74] Kadiyska T. Vassilev D. Tourtourikov I. Ciurinskiene S. Madzharova D. Savcheva M. . (2025). Age-dependent gut microbiome dysbiosis in autism spectrum disorder and the role of key bacterial ratios. Nutrients 17, 1775. doi: 10.3390/nu17111775, PMID: 40507042 PMC12157781

[B75] Kang D. W. Ilhan Z. E. Isern N. G. Hoyt D. W. Howsmon D. P. Shaffer M. . (2018). Differences in fecal microbial metabolites and microbiota of children with autism spectrum disorders. Anaerobe 49, 121–131. doi: 10.1016/j.anaerobe.2017.12.007, PMID: 29274915

[B76] Kang D. W. Adams J. B. Coleman D. M. Pollard E. L. Maldonado J. McDonough-Means S. . (2019). Long-term benefit of Microbiota Transfer Therapy on autism symptoms and gut microbiota. Sci. Rep. 9, 5821. doi: 10.1038/s41598-019-42183-0, PMID: 30967657 PMC6456593

[B77] Kantak P. A. Bobrow D. N. Nyby J. G. (2014). Obsessive-compulsive-like behaviors in house mice are attenuated by a probiotic (Lactobacillus rhamnosus GG). Behav. Pharmacol. 25, 71–79. doi: 10.1097/FBP.0000000000000013, PMID: 24257436

[B78] Kelly J. R. Kennedy P. J. Cryan J. F. Dinan T. G. Clarke G. Hyland N. P. . (2015). Breaking down the barriers: the gut microbiome, intestinal permeability and stress-related psychiatric disorders. Front. Cell Neurosci. 9, 392. doi: 10.3389/fncel.2015.00392, PMID: 26528128 PMC4604320

[B79] Kidd P. M. (2007). Omega-3 DHA and EPA for cognition, behavior, and mood: clinical findings and structural-functional synergies with cell membrane phospholipids. Altern. Med. Rev. 12, 207–227., PMID: 18072818

[B80] Kim J. W. Choi C. S. Kim K. C. Park J. H. Seung H. Joo S. H. . (2013). Gastrointestinal tract abnormalities induced by prenatal valproic Acid exposure in rat offspring. Toxicol. Res. 29, 173–179. doi: 10.5487/TR.2013.29.3.173, PMID: 24386517 PMC3877996

[B81] Kim S. Kim H. Yim Y. S. Ha S. Atarashi K. Tan T. G. . (2017). Maternal gut bacteria promote neurodevelopmental abnormalities in mouse offspring. Nature 549, 528–532. doi: 10.1038/nature23910, PMID: 28902840 PMC5870873

[B82] Koyama R. Ikegaya Y. (2015). Microglia in the pathogenesis of autism spectrum disorders. Neurosci. Res. 100, 1–5. doi: 10.1016/j.neures.2015.06.005, PMID: 26116891

[B83] Kratsman N. Getselter D. Elliott E. (2016). Sodium butyrate attenuates social behavior deficits and modifies the transcription of inhibitory/excitatory genes in the frontal cortex of an autism model. Neuropharmacology 102, 136–145. doi: 10.1016/j.neuropharm.2015.11.003, PMID: 26577018

[B84] Kreider C. M. Mburu S. Dizdarevic S. Garvan G. Elder J. H. (2021). Exploration of relationships among clinical gastrointestinal indicators and social and sensory symptom severity in children with autism spectrum disorder. Pediatr. Rep. 13, 594–604. doi: 10.3390/pediatric13040071, PMID: 34842807 PMC8628911

[B85] Lagod P. P. Naser S. A. (2023). The role of short-chain fatty acids and altered microbiota composition in autism spectrum disorder: A comprehensive literature review. Int. J. Mol. Sci. 24, 17432. doi: 10.3390/ijms242417432, PMID: 38139261 PMC10743890

[B86] Li X. Chauhan A. Sheikh A. M. Patil S. Chauhan V. Li X. M. . (2009). Elevated immune response in the brain of autistic patients. J. Neuroimmunol 207, 111–116. doi: 10.1016/j.jneuroim.2008.12.002, PMID: 19157572 PMC2770268

[B87] Li N. Yang J. Zhang J. Liang C. Wang Y. Chen B. . (2019). Correlation of gut microbiome between ASD children and mothers and potential biomarkers for risk assessment. Genomics Proteomics Bioinf. 17, 26–38. doi: 10.1016/j.gpb.2019.01.002, PMID: 31026579 PMC6520911

[B88] Li N. Chen H. Cheng Y. Xu F. Ruan G. Ying S. . (2021). Fecal microbiota transplantation relieves gastrointestinal and autism symptoms by improving the gut microbiota in an open-label study. Front. Cell Infect. Microbiol. 11, 759435. doi: 10.3389/fcimb.2021.759435, PMID: 34737978 PMC8560686

[B89] Li Y. Hu W. Lin B. Ma T. Zhang Z. Hu W. . (2024). Omic characterizing and targeting gut dysbiosis in children with autism spectrum disorder: symptom alleviation through combined probiotic and medium-carbohydrate diet intervention - a pilot study. Gut Microbes 16, 2434675–2434675. doi: 10.1080/19490976.2024.2434675, PMID: 39632378 PMC11622613

[B90] Liang X. Li R. Wong S. H. S. Sum R. K. W. Wang P. Yang B. . (2022). The effects of exercise interventions on executive functions in children and adolescents with autism spectrum disorder: A systematic review and meta-analysis. Sports Med. 52, 75–88. doi: 10.1007/s40279-021-01545-3, PMID: 34468951

[B91] Lim C. K. Essa M. M. de Paula M. R. Lovejoy D. B. Bilgin A. A. Waly M. I. . (2016). Altered kynurenine pathway metabolism in autism: Implication for immune-induced glutamatergic activity. Autism Res. 9, 621–631. doi: 10.1002/aur.1565, PMID: 26497015

[B92] Liu S. Li E. Sun Z. Fu D. Duan G. Jiang M. . (2019). Altered gut microbiota and short chain fatty acids in Chinese children with autism spectrum disorder. Sci. Rep. 9, 287. doi: 10.1038/s41598-018-36430-z, PMID: 30670726 PMC6342986

[B93] Liu J. Gao Z. Liu C. Liu T. Gao J. Cai Y. . (2022). Alteration of gut microbiota: new strategy for treating autism spectrum disorder. Front. Cell Dev. Biol. 10, 792490. doi: 10.3389/fcell.2022.792490, PMID: 35309933 PMC8929512

[B94] Ma B. Liang J. Dai M. Wang J. Luo J. Zhang Z. . (2019). Altered gut microbiota in chinese children with autism spectrum disorders. Front. Cell Infect. Microbiol. 9, 40. doi: 10.3389/fcimb.2019.00040, PMID: 30895172 PMC6414714

[B95] Macfabe D. F. (2012). Short-chain fatty acid fermentation products of the gut microbiome: implications in autism spectrum disorders. Microb. Ecol. Health Dis. 23. doi: 10.3402/mehd.v23i0.19260, PMID: 23990817 PMC3747729

[B96] MacFabe D. F. Cain D. P. Rodriguez-Capote K. Franklin A. E. Hoffman J. E. Boon F. . (2007). Neurobiological effects of intraventricular propionic acid in rats: possible role of short chain fatty acids on the pathogenesis and characteristics of autism spectrum disorders. Behav. Brain Res. 176, 149–169. doi: 10.1016/j.bbr.2006.07.025, PMID: 16950524

[B97] Mandy W. Lai M. C. (2016). Annual Research Review: The role of the environment in the developmental psychopathology of autism spectrum condition. J. Child Psychol. Psychiatry 57, 271–292. doi: 10.1111/jcpp.12501, PMID: 26782158

[B98] Manzari N. Matvienko-Sikar K. Baldoni F. O'Keeffe G. W. Khashan A. S. (2019). Prenatal maternal stress and risk of neurodevelopmental disorders in the offspring: a systematic review and meta-analysis. Soc. Psychiatry Psychiatr. Epidemiol. 54, 1299–1309. doi: 10.1007/s00127-019-01745-3, PMID: 31324962

[B99] Margolis K. G. Cryan J. F. Mayer E. A. (2021). The microbiota-gut-brain axis: from motility to mood. Gastroenterology 160, 1486–1501. doi: 10.1053/j.gastro.2020.10.066, PMID: 33493503 PMC8634751

[B100] Marler S. Ferguson B. J. Lee E. B. Peters B. Williams K. C. McDonnell E. . (2017). Association of rigid-compulsive behavior with functional constipation in autism spectrum disorder. J. Autism Dev. Disord. 47, 1673–1681. doi: 10.1007/s10803-017-3084-6, PMID: 28289979 PMC5526215

[B101] Martin C. R. Osadchiy V. Kalani A. Mayer E. A. (2018). The brain-gut-microbiome axis. Cell Mol. Gastroenterol. Hepatol. 6, 133–148. doi: 10.1016/j.jcmgh.2018.04.003, PMID: 30023410 PMC6047317

[B102] Mathew N. E. McCaffrey D. Walker A. K. Mallitt K. A. Masi A. Morris M. J. . (2024). The search for gastrointestinal inflammation in autism: a systematic review and meta-analysis of non-invasive gastrointestinal markers. Mol. Autism 15, 4. doi: 10.1186/s13229-023-00575-0, PMID: 38233886 PMC10795298

[B103] Mawe G. M. Hoffman J. M. (2013). Serotonin signalling in the gut–functions, dysfunctions and therapeutic targets. Nat. Rev. Gastroenterol. Hepatol. 10, 473–486. doi: 10.1038/nrgastro.2013.105, PMID: 23797870 PMC4048923

[B104] Messaoudi M. Lalonde R. Violle N. Javelot H. Desor D. Nejdi A. . (2011). Assessment of psychotropic-like properties of a probiotic formulation (Lactobacillus helveticus R0052 and Bifidobacterium longum R0175) in rats and human subjects. Br. J. Nutr. 105, 755–764. doi: 10.1017/S0007114510004319, PMID: 20974015

[B105] Mitsui R. Ono S. Karaki S. Kuwahara A. (2005). Neural and non-neural mediation of propionate-induced contractile responses in the rat distal colon. Neurogastroenterol Motil. 17, 585–594. doi: 10.1111/j.1365-2982.2005.00669.x, PMID: 16078948

[B106] Muller C. L. Anacker A. Veenstra-VanderWeele J. (2016). The serotonin system in autism spectrum disorder: From biomarker to animal models. Neuroscience 321, 24–41. doi: 10.1016/j.neuroscience.2015.11.010, PMID: 26577932 PMC4824539

[B107] Nankova B. B. Agarwal R. MacFabe D. F. La Gamma E. F. (2014). Enteric bacterial metabolites propionic and butyric acid modulate gene expression, including CREB-dependent catecholaminergic neurotransmission, in PC12 cells–possible relevance to autism spectrum disorders. PloS One 9, e103740. doi: 10.1371/journal.pone.0103740, PMID: 25170769 PMC4149359

[B108] Needham B. D. Adame M. D. Serena G. Rose D. R. Preston G. M. Conrad M. C. . (2021). Plasma and fecal metabolite profiles in autism spectrum disorder. Biol. Psychiatry 89, 451–462. doi: 10.1016/j.biopsych.2020.09.025, PMID: 33342544 PMC7867605

[B109] Needham B. D. Funabashi M. Adame M. D. Wang Z. Boktor J. C. Haney J. . (2022). A gut-derived metabolite alters brain activity and anxiety behaviour in mice. Nature 602, 647–653. doi: 10.1038/s41586-022-04396-8, PMID: 35165440 PMC9170029

[B110] Novau-Ferré N. Papandreou C. Rojo-Marticella M. Canals-Sans J. Bulló M. (2025). Gut microbiome differences in children with Attention Deficit Hyperactivity Disorder and Autism Spectrum Disorder and effects of probiotic supplementation: A randomized controlled trial. Res. Dev. Disabil. 161, 105003–105003. doi: 10.1016/j.ridd.2025.105003, PMID: 40184961

[B111] Obrenovich M. (2018). Leaky gut, leaky brain? Microorganisms 6, 107. doi: 10.3390/microorganisms6040107, PMID: 30340384 PMC6313445

[B112] Onore C. Careaga M. Ashwood P. (2012). The role of immune dysfunction in the pathophysiology of autism. Brain Behav. Immun. 26, 383–392. doi: 10.1016/j.bbi.2011.08.007, PMID: 21906670 PMC3418145

[B113] Panelli S. Capelli E. Lupo G. F. D. Schiepatti A. Betti E. Sauta E. . (2020). Comparative study of salivary, duodenal, and fecal microbiota composition across adult celiac disease. J. Clin. Med. 9, 1109. doi: 10.3390/jcm9041109, PMID: 32294965 PMC7231226

[B114] Paolicelli R. C. Bolasco G. Pagani F. Maggi L. Scianni M. Panzanelli P. . (2011). Synaptic pruning by microglia is necessary for normal brain development. Science 333, 1456–1458. doi: 10.1126/science.1202529, PMID: 21778362

[B115] Pascucci T. Colamartino M. Fiori E. Sacco R. Coviello A. Ventura R. . (2020). P-cresol alters brain dopamine metabolism and exacerbates autism-like behaviors in the BTBR mouse. Brain Sci. 10, 233. doi: 10.3390/brainsci10040233, PMID: 32294927 PMC7226382

[B116] Peretti S. Mariano M. Mazzocchetti C. Mazza M. Pino M C. Verrotti D. P. A. . (2019). Diet: the keystone of autism spectrum disorder? Nutr. Neurosci. 22, 825–839. doi: 10.1080/1028415X.2018.1464819, PMID: 29669486

[B117] Persico A. M. Napolioni V. (2013). Urinary p-cresol in autism spectrum disorder. Neurotoxicol Teratol 36, 82–90. doi: 10.1016/j.ntt.2012.09.002, PMID: 22975621

[B118] Pulikkan J. Maji A. Dhakan D. B. Saxena R. Mohan B. Anto M. M. . (2018). Gut microbial dysbiosis in Indian children with autism spectrum disorders. Microb. Ecol. 76, 1102–1114. doi: 10.1007/s00248-018-1176-2, PMID: 29564487

[B119] Saghazadeh A. Ataeinia B. Keynejad K. Abdolalizadeh A. Hirbod-Mobarakeh A. Rezaei N. . (2019). Anti-inflammatory cytokines in autism spectrum disorders: A systematic review and meta-analysis. Cytokine 123, 154740. doi: 10.1016/j.cyto.2019.154740, PMID: 31228728

[B120] Santocchi E. Guiducci L. Fulceri F. Billeci L. Buzzigoli E. Apicella F. . (2016). Gut to brain interaction in Autism Spectrum Disorders: a randomized controlled trial on the role of probiotics on clinical, biochemical and neurophysiological parameters. BMC Psychiatry 16, 183. doi: 10.1186/s12888-016-0887-5, PMID: 27260271 PMC4893248

[B121] Savitz J. Drevets W. C. Wurfel B. E. Ford B. N. Bellgowan P. S. Victor T. A. . (2015). Reduction of kynurenic acid to quinolinic acid ratio in both the depressed and remitted phases of major depressive disorder. Brain Behav. Immun. 46, 55–59. doi: 10.1016/j.bbi.2015.02.007, PMID: 25686798 PMC4414807

[B122] Schmitz O. S. McFadden B. A. Golem D. L. Pellegrino J. K. Walker A. J. Sanders D. J. . (2017). The effects of exercise dose on stereotypical behavior in children with autism. Med. Sci. Sports Exerc 49, 983–990. doi: 10.1249/MSS.0000000000001197, PMID: 28060033

[B123] Settanni C. R. Bibbo S. Ianiro G. Rinninella E. Cintoni M. Mele M. C. . (2021). Gastrointestinal involvement of autism spectrum disorder: focus on gut microbiota. Expert Rev. Gastroenterol. Hepatol. 15, 599–622. doi: 10.1080/17474124.2021.1869938, PMID: 33356668

[B124] Sgritta M. Dooling S. W. Buffington S. A. Momin E. N. Francis M. B. Britton R. A. . (2019). Mechanisms underlying microbial-mediated changes in social behavior in mouse models of autism spectrum disorder. Neuron 101, 246–259.e6. doi: 10.1016/j.neuron.2018.11.018, PMID: 30522820 PMC6645363

[B125] Shaaban S. Y. El-Gendy Y. G. Mehanna N. S. El-Senousy W. M. El-Feki H. S. A. Saad K. . (2018). The role of probiotics in children with autism spectrum disorder: A prospective, open-label study. Nutr. Neurosci. 21, 676–681. doi: 10.1080/1028415X.2017.1347746, PMID: 28686541

[B126] Sharon G. Cruz N. J. Kang D. W. Gandal M. J. Wang B. Kim Y. M. . (2019). Human gut microbiota from autism spectrum disorder promote behavioral symptoms in mice. Cell 177, 1600–1618.e17. doi: 10.1016/j.cell.2019.05.004, PMID: 31150625 PMC6993574

[B127] Silva Y. P. Bernardi A. Frozza R. L. (2020). The role of short-chain fatty acids from gut microbiota in gut-brain communication. Front. Endocrinol. (Lausanne) 11, 25. doi: 10.3389/fendo.2020.00025, PMID: 32082260 PMC7005631

[B128] Singh S. Yazdani U. Gadad B. Zaman S. Hynan L. S. Roatch N. . (2017). Serum thyroid-stimulating hormone and interleukin-8 levels in boys with autism spectrum disorder. J. Neuroinflamm. 14, 113. doi: 10.1186/s12974-017-0888-4, PMID: 28577577 PMC5457729

[B129] Slattery J. A. Page A. J. Dorian C. L. Brierley S. M. Blackshaw L. A. (2006). Potentiation of mouse vagal afferent mechanosensitivity by ionotropic and metabotropic glutamate receptors. J. Physiol. 577, 295–306. doi: 10.1113/jphysiol.2006.117762, PMID: 16945965 PMC2000674

[B130] Socala K. Doboszewska U. Szopa A. Serefko A. Wlodarczyk M. Zielinska A. . (2021). The role of microbiota-gut-brain axis in neuropsychiatric and neurological disorders. Pharmacol. Res. 172, 105840. doi: 10.1016/j.phrs.2021.105840, PMID: 34450312

[B131] Spratt E. G. Nicholas J. S. Brady K. T. Carpenter L. A. Hatcher C. R. Meekins K. A. . (2012). Enhanced cortisol response to stress in children in autism. J. Autism Dev. Disord. 42, 75–81. doi: 10.1007/s10803-011-1214-0, PMID: 21424864 PMC3245359

[B132] Stewart C. A. Needham B. D. Meyer C. R. Tan J. Conrad M. Preston G. M. . (2022). Safety and target engagement of an oral small-molecule sequestrant in adolescents with autism spectrum disorder: an open-label phase 1b/2a trial. Nat. Med. 28, 528–534. doi: 10.1038/s41591-022-01683-9, PMID: 35165451

[B133] Stilling R. M. van de Wouw M. Clarke G. Stanton C. Dinan T. G. Cryan J. F. . (2016). The neuropharmacology of butyrate: The bread and butter of the microbiota-gut-brain axis? Neurochem. Int. 99, 110–132. doi: 10.1016/j.neuint.2016.06.011, PMID: 27346602

[B134] Stilling R. M. Moloney G. M. Ryan F. J. Hoban A. E. Bastiaanssen T. F. Shanahan F. . (2018). Social interaction-induced activation of RNA splicing in the amygdala of microbiome-deficient mice. Elife 7, e33070. doi: 10.7554/eLife.33070.025, PMID: 29809134 PMC5995540

[B135] Sudo N. Chida Y. Aiba Y. Sonoda J. Oyama N. Yu X. N. . (2004). Postnatal microbial colonization programs the hypothalamic-pituitary-adrenal system for stress response in mice. J. Physiol. 558, 263–275. doi: 10.1113/jphysiol.2004.063388, PMID: 15133062 PMC1664925

[B136] Sullivan E. L. Nousen E. K. Chamlou K. A. (2014). Maternal high fat diet consumption during the perinatal period programs offspring behavior. Physiol. Behav. 123, 236–242. doi: 10.1016/j.physbeh.2012.07.014, PMID: 23085399 PMC3594403

[B137] Suren P. Roth C. Bresnahan M. Haugen M. Hornig M. Hirtz D. . (2013). Association between maternal use of folic acid supplements and risk of autism spectrum disorders in children. JAMA 309, 570–577. doi: 10.1001/jama.2012.155925, PMID: 23403681 PMC3908544

[B138] Swanson K. S. Gibson G. R. Hutkins R. Reimer R. A. Reid G. Verbeke K. . (2020). The International Scientific Association for Probiotics and Prebiotics (ISAPP) consensus statement on the definition and scope of synbiotics. Nat. Rev. Gastroenterol. Hepatol. 17, 687–701. doi: 10.1038/s41575-020-0344-2, PMID: 32826966 PMC7581511

[B139] Toscano C. Carvalho H. M. Ferreira J. P. (2018). Exercise effects for children with autism spectrum disorder: metabolic health, autistic traits, and quality of life. Percept. Mot Skills 125, 126–146. doi: 10.1177/0031512517743823, PMID: 29226773

[B140] Vargas D. L. Nascimbene C. Krishnan C. Zimmerman A. W. Pardo C. A. (2005). Neuroglial activation and neuroinflammation in the brain of patients with autism. Ann. Neurol. 57, 67–81. doi: 10.1002/ana.20315, PMID: 15546155

[B141] Vuong H. E. Hsiao E. Y. (2017). Emerging roles for the gut microbiome in autism spectrum disorder. Biol. Psychiatry 81, 411–423. doi: 10.1016/j.biopsych.2016.08.024, PMID: 27773355 PMC5285286

[B142] Wang L. Christophersen C. T. Sorich M. J. Gerber J. P. Angley M. T. Conlon M. A. (2012). Elevated fecal short chain fatty acid and ammonia concentrations in children with autism spectrum disorder. Dig Dis. Sci. 57, 2096–2102. doi: 10.1007/s10620-012-2167-7, PMID: 22535281

[B143] Wang M. Wan J. Rong H. He F. Wang H. Zhou J. . (2019). Alterations in gut glutamate metabolism associated with changes in gut microbiota composition in children with autism spectrum disorder. mSystems 4, e00321–18. doi: 10.1128/msystems.00321-18, PMID: 30701194 PMC6351726

[B144] Wang Y. Li N. Yang J. J. Zhao D. M. Chen B. Zhang G. Q. . (2020). Probiotics and fructo-oligosaccharide intervention modulate the microbiota-gut brain axis to improve autism spectrum reducing also the hyper-serotonergic state and the dopamine metabolism disorder. Pharmacol. Res. 157, 104784. doi: 10.1016/j.phrs.2020.104784, PMID: 32305492

[B145] Wang J. Ma B. Wang J. Zhang Z. Chen O. (2022). Global prevalence of autism spectrum disorder and its gastrointestinal symptoms: A systematic review and meta-analysis. Front. Psychiatry 13, 963102. doi: 10.3389/fpsyt.2022.963102, PMID: 36081466 PMC9445193

[B146] Wenzel T. J. Gates E. J. Ranger A. L. Klegeris A. (2020). Short-chain fatty acids (SCFAs) alone or in combination regulate select immune functions of microglia-like cells. Mol. Cell Neurosci. 105, 103493. doi: 10.1016/j.mcn.2020.103493, PMID: 32333962

[B147] Whiteley P. Haracopos D. Knivsberg A. M. Reichelt K. L. Parlar S. Jacobsen J. . (2010). The ScanBrit randomised, controlled, single-blind study of a gluten- and casein-free dietary intervention for children with autism spectrum disorders. Nutr. Neurosci. 13, 87–100. doi: 10.1179/147683010X12611460763922, PMID: 20406576

[B148] Williams J. A. Burgess S. Suckling J. Lalousis P. A. Batool F. Griffiths S. L. . (2022). Inflammation and brain structure in schizophrenia and other neuropsychiatric disorders: A mendelian randomization study. JAMA Psychiatry 79, 498–507. doi: 10.1001/jamapsychiatry.2022.0407, PMID: 35353173 PMC8968718

[B149] Worsham W. Dalton S. Bilder D. A. (2021). The prenatal hormone milieu in autism spectrum disorder. Front. Psychiatry 12, 655438. doi: 10.3389/fpsyt.2021.655438, PMID: 34276434 PMC8280339

[B150] Wu W. L. Adame M. D. Liou C. W. Barlow J. T. Lai T. T. Sharon G. . (2021). Microbiota regulate social behaviour *via* stress response neurons in the brain. Nature 595, 409–414. doi: 10.1038/s41586-021-03669-y, PMID: 34194038 PMC8346519

[B151] Xiao L. Yan J. Yang T. Zhu J. Li T. Wei H. . (2021). Fecal microbiome transplantation from children with autism spectrum disorder modulates tryptophan and serotonergic synapse metabolism and induces altered behaviors in germ-free mice. mSystems 6, e01343–20. doi: 10.1128/msystems.01343-20, PMID: 33824200 PMC8547010

[B152] Xue Y. An S. Qiu W. Zhang W. Fu L. Zhen Z. . (2023). Exercise changes gut microbiota: A new idea to explain that exercise improves autism. Int. J. Sports Med. 44, 473–483. doi: 10.1055/a-2018-2477, PMID: 36690029

[B153] Yildirim V. Simsek S. Cetin I. Dokuyucu R. (2023). Kynurenine, kynurenic acid, quinolinic acid and interleukin-6 levels in the serum of patients with autism spectrum disorder. Med. (Kaunas) 59, 1906. doi: 10.3390/medicina59111906, PMID: 38003955 PMC10673218

[B154] Yu C. C. W. Wong S. W. L. Lo F. S. F. So R. C. H. Chan D. F. Y. (2018). Study protocol: a randomized controlled trial study on the effect of a game-based exercise training program on promoting physical fitness and mental health in children with autism spectrum disorder. BMC Psychiatry 18, 56. doi: 10.1186/s12888-018-1635-9, PMID: 29486750 PMC5830347

[B155] Yu C. D. Xu Q. J. Chang R. B. (2020). Vagal sensory neurons and gut-brain signaling. Curr. Opin. Neurobiol. 62, 133–140. doi: 10.1016/j.conb.2020.03.006, PMID: 32380360 PMC7560965

[B156] Zeidan J. Fombonne E. Scorah J. Ibrahim A. Durkin M. S. Saxena S. . (2022). Global prevalence of autism: A systematic review update. Autism Res. 15, 778–790. doi: 10.1002/aur.2696, PMID: 35238171 PMC9310578

[B157] Zhang M. Ma W. Zhang J. He Y. Wang J. (2018). Analysis of gut microbiota profiles and microbe-disease associations in children with autism spectrum disorders in China. Sci. Rep. 8, 13981. doi: 10.1038/s41598-018-32219-2, PMID: 30228282 PMC6143520

[B158] Zhang L. Bang S. He Q. Matsuda M. Luo X. Jiang Y. H. . (2023). SHANK3 in vagal sensory neurons regulates body temperature, systemic inflammation, and sepsis. Front. Immunol. 14, 1124356. doi: 10.3389/fimmu.2023.1124356, PMID: 36845137 PMC9944123

[B159] Zheng Y. Bek M. K. Prince N. Z. Peralta M. Garssen J. Perez P. P. . (2021). The role of bacterial-derived aromatic amino acids metabolites relevant in autism spectrum disorders: A comprehensive review. Front. Neurosci. 15, 738220. doi: 10.3389/fnins.2021.738220, PMID: 34744609 PMC8568365

[B160] Zhou H. Xu X. Yan W. Zou X. Wu L. Luo X. . (2020). Prevalence of autism spectrum disorder in China: A nationwide multi-center population-based study among children aged 6 to 12 years. Neurosci. Bull. 36, 961–971. doi: 10.1007/s12264-020-00530-6, PMID: 32607739 PMC7475160

